# The MYO6 interactome reveals adaptor complexes coordinating early endosome and cytoskeletal dynamics

**DOI:** 10.15252/embr.201744884

**Published:** 2018-02-21

**Authors:** Thomas O'Loughlin, Thomas A Masters, Folma Buss

**Affiliations:** ^1^ Cambridge Institute for Medical Research Wellcome Trust/MRC Building Cambridge UK

**Keywords:** BioID, endosome, functional proteomics, interactome, MYO6, Cell Adhesion, Polarity & Cytoskeleton, Membrane & Intracellular Transport, Methods & Resources

## Abstract

The intracellular functions of myosin motors requires a number of adaptor molecules, which control cargo attachment, but also fine‐tune motor activity in time and space. These motor–adaptor–cargo interactions are often weak, transient or highly regulated. To overcome these problems, we use a proximity labelling‐based proteomics strategy to map the interactome of the unique minus end‐directed actin motor MYO6. Detailed biochemical and functional analysis identified several distinct MYO6‐adaptor modules including two complexes containing RhoGEFs: the LIFT (LARG‐Induced F‐actin for Tethering) complex that controls endosome positioning and motility through RHO‐driven actin polymerisation; and the DISP (DOCK7‐Induced Septin disPlacement) complex, a novel regulator of the septin cytoskeleton. These complexes emphasise the role of MYO6 in coordinating endosome dynamics and cytoskeletal architecture. This study provides the first *in vivo* interactome of a myosin motor protein and highlights the power of this approach in uncovering dynamic and functionally diverse myosin motor complexes.

## Introduction

In eukaryotic cells, myosin motor proteins regulate the distribution of a wide variety of cytoplasmic cargo by mediating short‐range transport or tethering of organelles, vesicles, mRNA and protein complexes. The myosin superfamily can be grouped into at least 35 different classes, which all translocate towards the plus‐end of actin filaments with the exception of MYO6 1. The unique directionality of MYO6 facilitates its specific cellular roles in endocytosis, receptor trafficking, protein secretion and autophagy. Loss of these functions underlies a number of phenotypes observed in the MYO6‐null *Snell's waltzer* mouse, or in humans harbouring mutations in the MYO6 gene, including deafness, astrogliosis, proteinuria as well as hypertrophic cardiomyopathy 2, 3, 4, 5, 6. Furthermore, overexpression of MYO6 is a hallmark of a number of cancers including prostate cancer 7.

The functional and phenotypic diversity associated with MYO6 arises from interactions with multiple cargo adaptors including disabled‐2 (DAB2), GAIP‐interacting protein C‐terminus (GIPC1), target of Myb 1 (TOM1), lemur tyrosine kinase 2 (LMTK2), optineurin (OPTN), TAX1 binding protein 1 (TAX1BP1) and nuclear dot protein 52 (NDP52) 8, 9, 10, 11, 12, 13. These interactions occur at two major protein binding motifs, the RRL and WWY (named after their amino acid composition), which are located within two distinct subdomains of a unique C‐terminal cargo‐binding tail 10, 11. The tail also contains a phosphatidylinositol 4,5‐bisphosphate (PIP_2_) binding motif, which aids recruitment of the motor to membranes along with its binding partners 14. In addition, two distinct ubiquitin‐binding sites—a motif interacting with ubiquitin (MIU) and a MYO6 ubiquitin‐binding domain (MyUb)—in the tail region may bind ubiquitinated cargo or regulate other interactions 15, 16. These adaptor interactions mediate targeting of the motor to its appropriate cellular location, making them a critical determinant of motor function.

Interestingly, adaptor binding to the tail domain not only mediates cargo attachment but can also coordinate motor activity. In the case of MYO6, cargo binding can initiate unfolding, thereby releasing inhibition of motor activity 17. In addition, growing evidence suggests that the myosin tail region can also directly impact on actin filament dynamics: for example, myosins of class IX contain a tail domain with RhoGAP activity 18; MYO5A interacts with the actin nucleator SPIRE2 to coordinate actin polymerisation on RAB11 endosomes 19; and myosins of class I can interact with machinery that regulates the ARP2/3 complex, and thus actin remodelling, during endocytosis in both yeast and mammalian cells 20, 21, 22. Together, these findings highlight an emerging role for the myosin tail domain beyond simple cargo recognition, in modulating both motor activity and the actin track.

Traditional approaches such as yeast two‐hybrid, native immunoprecipitation as well as pull‐down assays with the cargo‐binding tail domain have thus far mainly uncovered only direct MYO6 cargo adaptors, but not organelle anchors or cargoes themselves, which might often comprise multi‐protein complexes. New approaches are thus required to identify weak, transient motor–cargo and motor–track interactions, which enable the spatial and temporal coordination of diverse MYO6 functions. Therefore, to uncover the larger MYO6 interaction network—the MYO6 interactome—we employed *in situ* proximity labelling by BioID to identify proteins that may enable the spatial and temporal regulation of cargo binding to MYO6 as well as its motor activity and actin track dynamics 23. This method utilises a promiscuous variant of the *E. coli* biotin ligase (BirA*) which releases a reactive biotin intermediate (biotinoyl‐5′‐AMP) into its surroundings 23, 24. Subsequently, biotinoyl‐5′‐AMP can react with primary amines in proximal proteins which can then be isolated using the high‐affinity interaction between the newly generated biotin tag and streptavidin. As the biotin is covalently attached to its target, this permits lysis and purification under harsh, denaturing conditions while still preserving weak or transient interactions.

This first *in vivo* proximity map of a myosin motor protein highlights the complex interactome and multi‐functionality of MYO6 and includes a new direct binding partner and at least four new multi‐protein complexes. We verified a large number of these network components by affinity pull‐down and functional analysis, thereby highlighting a potential role for MYO6 in coordinating actin dynamics with endosome function and septin filament positioning. This approach provides a powerful means of elucidating the dynamic subcomplexes formed by unconventional myosins to orchestrate diverse roles and coordinate motor activity with cytoskeletal dynamics.

## Results

### The MYO6 interactome reveals numerous novel binding partners

**Figure 1:**
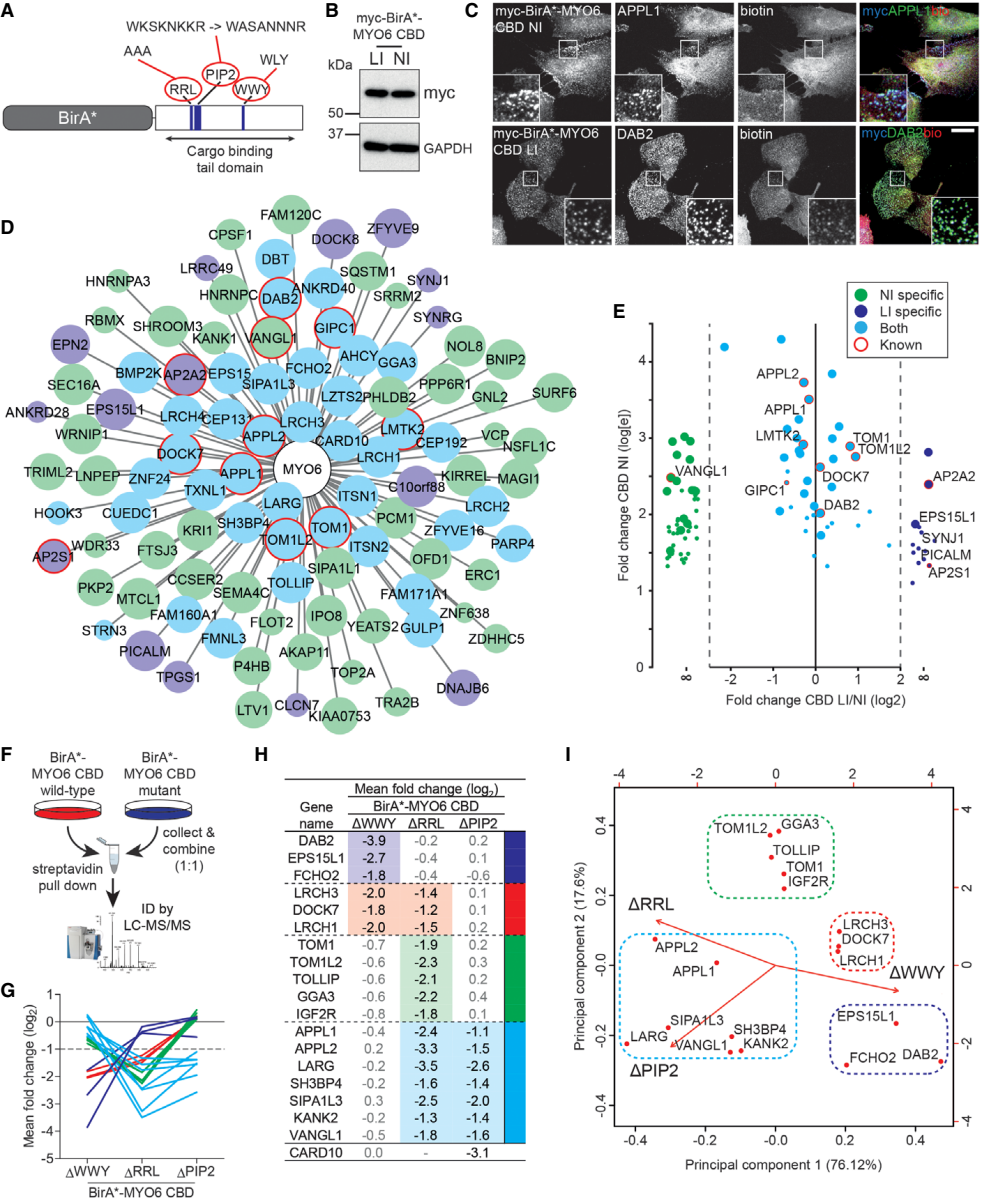
The MYO6 interactome reveals numerous novel binding partners Schematic diagram of BirA*‐MYO6 CBD wild‐type and mutant constructs.myc‐BirA*‐MYO6 CBD RPE cell lines were analysed by immunoblot using myc and GAPDH (loading control) antibodies.Immunofluorescence microscope images of RPE cells stably expressing myc‐BirA*‐MYO6 CBD NI (top row) and myc‐BirA*‐MYO6 CBD LI (bottom row) treated with 50 μM biotin for 24 h. Cells were immunostained with antibodies to myc (blue), APPL1 (green, top row), DAB2 (green, bottom row) or streptavidin (red) to visualise biotinylated proteins. Scale bar, 20 μm.Diagram of all direct and indirect interactions identified for MYO6 NI and LI using BioID. Edge length corresponds to FC‐A score (lower score = greater length) and node size to SAINT score (lower confidence = smaller node). Green and blue nodes indicate interactions specific to the NI and LI isoforms, respectively, and cyan indicates shared partners. Previously described interactions are highlighted by red outlines.Graph depicting relative enrichment (the fold‐change ratio) of proteins in pull‐downs from MYO6 NI and LI expressing cells. Dot size corresponds to SAINT score (lower confidence = smaller dot).Schematic diagram of SILAC workflow.Plot depicting mean log_2_ fold change for each protein in (H) across the different MYO6 mutants. Colours correspond to those in (H).Table of proteins identified with a significant (significance A, FDR < 5%) loss in at least one experiment. Heavy/light ratios are provided and > twofold losses are highlighted in colour. Proteins are grouped by colour based on pattern of loss across the different MYO6 mutants. − = not identified in the experiment.Plot of principal component analysis using the mean log_2_ fold change from triplicate experiments. The figure shows the projections of the data on the first (*x*‐axis) and second (*y*‐axis) principal components. Principal components 1–3 account for 76.12, 17.6 and 6.274% of the variability in the data, respectively. CARD10 was excluded from the analysis due to its absence from the ΔRRL data set. Dashed boxes highlight the clusters likely to represent distinct MYO6‐associated protein complexes. Colours correspond to those in (H).

To capture its dynamic, highly transient cargo interactions, we used *in situ* proximity labelling (BioID) to survey the MYO6 interactome in living cells under steady state conditions. We generated retinal pigment epithelial (RPE) cell lines stably expressing BirA*‐MYO6 fusion proteins of the cargo‐binding domain (CBD) derived from the “no insert” isoform (NI), which is targeted predominantly to early APPL1‐positive endosomes; or the “large insert” isoform (LI), which is targeted strongly to clathrin‐coated structures (CCS) positive for DAB2 (Fig 1A–C). As BioID has a limited labelling radius, we used the truncated CBD in our experiments which is sufficient for adaptor and lipid binding and therefore subcellular targeting.

We performed large‐scale streptavidin pull‐downs from BirA*, BirA*‐MYO6 CBD NI and BirA*‐MYO6 CBD LI RPE cell lines and identified enriched proteins by mass spectrometry (MS). BirA*‐MYO6 CBD replicates were analysed by MS and compared against a bank of 10 BirA* only negative control experiments using the online tool at CRAPome.org 25. Using a fold‐change (FC‐A) threshold of ≥ 3 and a Significance Analysis of INTeractome (SAINT) probability threshold of ≥ 0.8 26, we identified 102 high‐confidence proximal proteins. These included the majority of the known direct binding partners of MYO6 such as TOM1/L2, DAB2, GIPC1 and LMTK2, in addition to > 90 novel MYO6‐associated proteins, which might bind directly or as part of larger MYO6‐associated protein complexes (Fig 1D). Comparison of the NI and LI showed 39 shared interactions and 16 or 47 specific interactions for the LI and NI isoforms, respectively. Many of the known direct binding partners of MYO6 appear in the shared pool of interactions for the two isoforms (Fig 1E). This confirms our previous observations that binding of DAB2 and other adaptors is not isoform specific 8, 10, 14, but targeting of the LI isoform to clathrin‐coated structures is directed by the large insert 27. As a result, the LI still appears to show enrichment for CCS proteins such as AP2 subunits, SYNJ1 and PICALM, whereas the NI specific interactions are less well annotated but are likely to link it to diverse cellular localisations and functions.

### Mutational profiling identifies distinct MYO6‐associated protein complexes

**Figure EV1:**
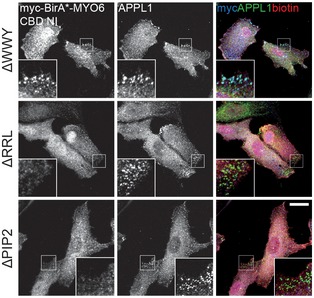
Characterisation of BirA*‐MYO6 CBD NI mutant cell lines Immunofluorescence microscope images of RPE cells stably expressing myc‐BirA*‐MYO6 CBD NI ΔWWY (top row), ΔRRL (middle row) and ΔPIP2 (bottom row) treated with 50 μM biotin for 24 h. Cells were immunostained with myc (blue) and APPL1 (green) antibodies. Scale bar, 20 μm.

To verify the novel MYO6 interaction network, we introduced mutations into the known cargo‐binding and lipid‐binding sites, the WWY, RRL and PIP_2_ binding motifs 10, 11, 14 and assessed differences in the relative abundance of BirA*‐MYO6 binding partners using a SILAC‐based approach (Fig 1F). Analysis of these cells by confocal microscopy also revealed that the ΔRRL and ΔPIP2, but not the ΔWWY mutation, perturbed MYO6 targeting to APPL1 endosomes (Fig EV1A). The different tail mutants caused loss of specific MYO6 binding partners; for example, mutation of the known DAB2 binding site, the WWY motif, led to the loss of DAB2 as well as other endocytosis‐associated proteins such as EPS15L1 and FCHO2 (Fig 1G and H). Alternatively, deletion of the RRL and PIP_2_ motifs caused reduced association of the early endosome proteins APPL1 and APPL2, and the RRL with TOM1/L2. Finally, LRCH3, LRCH1 and DOCK7 interactions were blocked by mutation of both the WWY and RRL motifs (Fig 1G and H). Use of principal component analysis to cluster the data according to shared behaviour across the mutants implied the existence of four independent MYO6‐associated protein complexes containing the known MYO6 binding partners DAB2, TOM1/L2, APPL1/2 and DOCK7, respectively (Fig 1I).

### The MYO6 interactome can be verified by secondary screens

**Figure 2:**
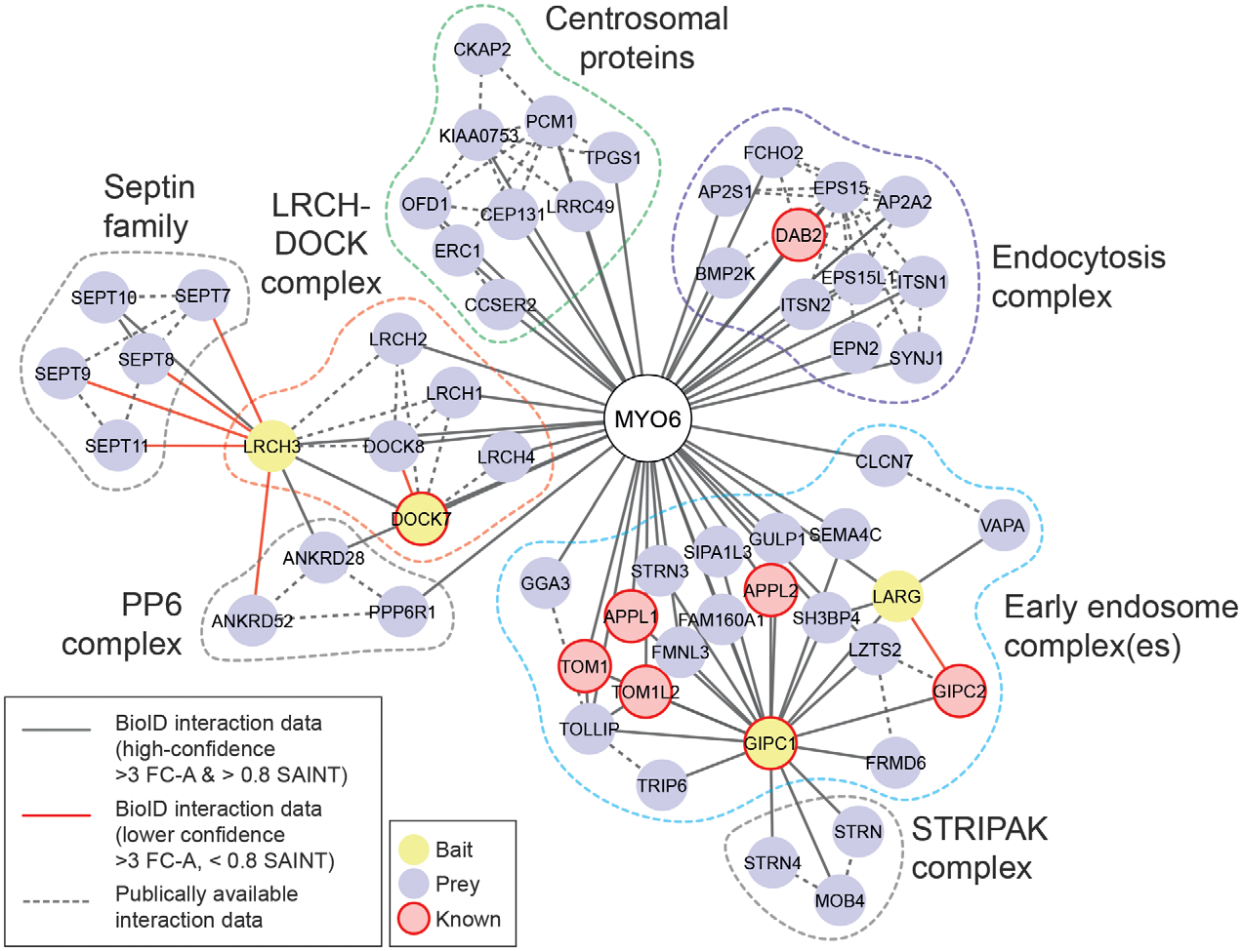
The MYO6 interactome can be verified by secondary screens Diagram of the MYO6 protein interaction network identified by BioID (solid lines) with MYO6 (white), GIPC1, LARG, LRCH3 and DOCK7 (yellow) baits and supplemented with interaction data available in public databases (dashed lines). Previously identified MYO6 binding partners are indicated in pink. Lower confidence interactions (> 3 FC‐A, < 0.8 SAINT) are indicated by red lines. All proteins < 2 interactions in the network were excluded for simplicity and further adjustments made manually. Proteins were clustered using a force‐directed layout function in Cytoscape 58. Putative complexes/subcellular locations are highlighted by the dashed boxes.

To further explore the composition of these MYO6‐associated complexes, we performed secondary BioID screens using four high‐confidence hits from the MYO6 interactome: GIPC1, the uncharacterised protein LRCH3 and the guanine nucleotide‐exchange factors (GEFs) DOCK7 and LARG. These screens with secondary baits identified a MYO6 interactome containing more than 130 proteins, summarised in the network in Fig 2. By combining our data with interaction data from public databases and using a semi‐supervised clustering approach, we uncovered distinct clusters corresponding to putative MYO6‐associated complexes. These data largely overlapped with the mutational profiling experiments, identifying: a complex containing DAB2 involved in clathrin‐mediated endocytosis; proteins such as GIPC1 and TOM1 associated with early endosomes; a multitude of proteins linking MYO6 to distinct functions at the plasma membrane or peripheral endosomes; and an uncharacterised complex composed of DOCK7 and multiple members of the leucine‐rich repeat and calponin homology domain‐containing (LRCH) protein family.

### GIPC1 links MYO6 to multiple protein complexes

**Figure 3:**
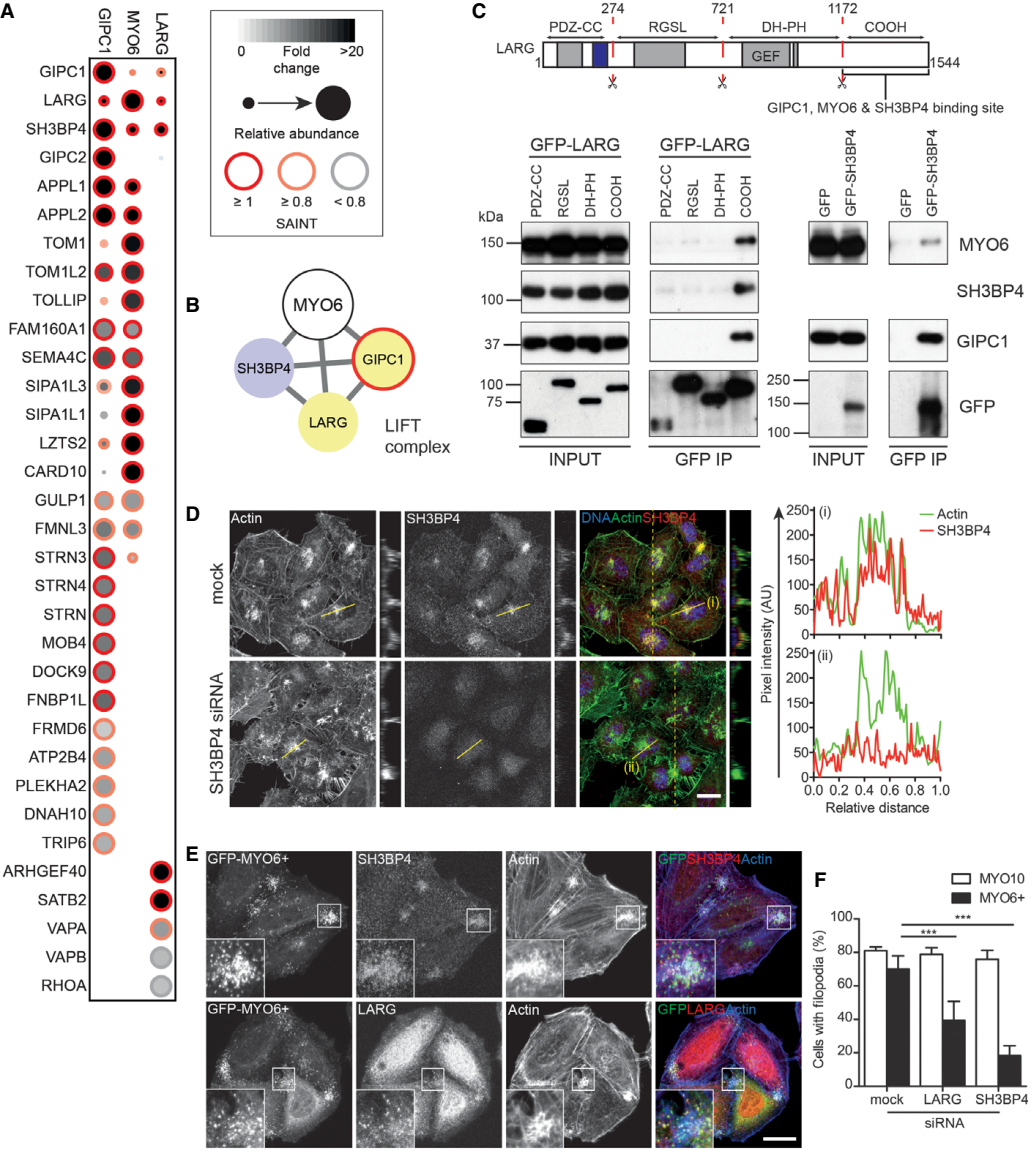
GIPC1 links MYO6 to multiple protein complexes Dot plot of high and medium confidence interactions (> 3 FC‐A and > 0.8 SAINT or > 3 FC‐A and < 0.8 SAINT, respectively) identified in BirA*‐GIPC1 and BirA*‐LARG experiments and shared interactors from the BirA*‐MYO6 CBD interactome.Network diagram of the LIFT complex.Top: Schematic cartoon of LARG domain structure. Bottom: GFP nanobody immunoprecipitates from HEK293T cells transfected with GFP and GFP‐tagged LARG fragments encompassing amino acids 1–274 (PDZ‐CC), 274–721 (RGSL), 721–1172 (DH‐PH) and 1171–1544 (COOH) (left) or GFP and full‐length GFP‐SH3BP4 (right). Samples were analysed by Western blot with the indicated antibodies.Confocal microscope images of SH3BP4 HeLa siRNA KD cells immunostained for SH3BP4 (red) and actin (green). Images are maximum intensity projections of confocal stacks. Views through the *z*‐stack (yellow dashed line) are shown. Scale bar, 20 μm. Graphs (i) and (ii) on the right: pixel intensity profiles of SH3BP4 and actin labelling along yellow line.Confocal microscope images of HeLa cells transiently transfected with GFP‐MYO6^+^ (green) and immunostained for SH3BP4 (top row, red), LARG (bottom row, red) and actin (blue). Images are maximum intensity projections of confocal stacks. Scale bar, 20 μm.Graph depicting the mean percentage of MYO6^+^ or MYO10‐positive cells treated with mock, LARG or SH3BP4 siRNA which generated filopodia. Counts were performed on cells from 10 fields of view (typically 10–30 cells/field) and *n* = 3 independent experiments. A two‐sample *t*‐test was used to determine statistical significance. ***P < 0.001. Error bars indicate SEM.

Mapping of the GIPC1 interactome using BioID identified 25 high‐confidence interaction candidates including multiple members of the signalling and scaffold complex STRIPAK (MOB4, STRN, STRN3 and STRN4) 28 (Figs 2 and 3A). The GIPC1 and MYO6 data sets contained 14 mutual interactions including known links such as APPL1/2 and several novel shared associations such as GULP1, LZTS2, FAM160A1, SIPA1L1/3 and FMNL3 (Fig 3A). Of particular interest were LARG, SH3BP4 and CARD10, which were extremely high‐confidence MYO6 interactors, and were also present in the GIPC1 data set to varying degrees. BirA*‐LARG pull‐downs revealed five high‐confidence interactions, which included both GIPC1 and SH3BP4 (Figs 2 and 3A).

**Figure EV2:**
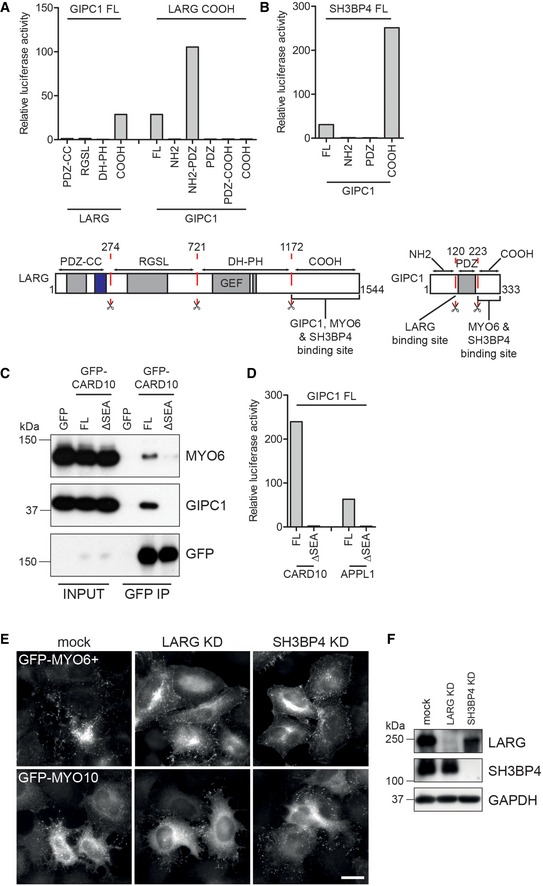
GIPC1 links MYO6 to multiple protein complexes A, BTop: The mammalian two‐hybrid assay was used to test binding of (A) full‐length GIPC1 against fragments of LARG encompassing amino acids 1–274 (PDZ‐CC), 274–721 (RGSL), 721–1172 (DH‐PH) and 1171–1544 (COOH) or LARG COOH against fragments GIPC1 encompassing amino acids 1–120 (NH2), 1–223 (NH2‐PDZ), 120–223 (PDZ), 120–333 (PDZ‐COOH), 223–333 (COOH) and (B) full‐length SH3BP4 against fragments GIPC1 encompassing amino acids 1–120 (NH2), 120–223 (PDZ), 223–333 (COOH). Graphs show the mean relative luciferase activity from single representative experiments. Bottom: Schematic cartoon highlighting domain structure, fragments and binding sites found in LARG (left) and GIPC1 (right).CGFP nanobody immunoprecipitates from HEK293T cells transfected with GFP and GFP‐tagged CARD10 full length or missing the C‐terminal four amino acids (ΔSEA). Samples were analysed by Western blot with the indicated antibodies.DThe mammalian two‐hybrid assay was used to test binding of full‐length CARD10 and CARD10 ΔSEA to full‐length GIPC1. Graphs show the mean relative luciferase activity from single representative experiments.EWidefield microscope images of HeLa cells transfected with GFP‐MYO6^+^ or GFP‐MYO10 and treated with mock, LARG or SH3BP4 siRNA. Cells were immunostained with a GFP antibody. Scale bar, 20 μm.FHeLa cells treated with mock, LARG or SH3BP4 siRNA were analysed by Western blot using LARG, SH3BP4 and GAPDH (loading control) antibodies.

We next validated these putative MYO6‐GIPC1 complexes using complementary methodologies. Full‐length SH3BP4 and domain fragments of LARG were tested for binding by immunoprecipitation (IP) from HEK293T cells or for direct interactions using the mammalian two‐hybrid (M2H) assay. These experiments confirmed the existence of a complex consisting of MYO6, GIPC1, LARG and SH3BP4 (Fig 3B). The C‐terminal fragment of LARG (amino acids 1171–1544) co‐immunoprecipitated GIPC1, MYO6 and SH3BP4, and appears to be incorporated into the complex through an interaction with the N‐terminal end of the PDZ domain of GIPC1 (Figs 3C and EV2A). SH3BP4 also co‐immunoprecipitated GIPC1 and MYO6, and engaged the complex through the C‐terminal fragment of GIPC1, much like MYO6 (Figs 3C and EV2B). As SH3BP4 successfully co‐immunoprecipitated MYO6, it appears they can bind the C‐terminal region of GIPC1 simultaneously.

We observed that CARD10 contains a C‐terminal PDZ binding motif, SEA, which is identical to the motif present in APPL1 and is required for its interaction with GIPC1, but not found in other CARD proteins (CARD11 and CARD14). IPs from HEK293T cell expressing GFP‐tagged full‐length CARD10 confirmed binding to GIPC1 and MYO6; however, deletion of the SEA motif completely abolished binding (Fig EV2C). As expected, CARD10 bound directly to full‐length GIPC1 via its C‐terminal SEA motif in the M2H assay (Fig EV2D).

Together, these data show that, in addition to its known interactions with APPL1 and VANGL1 29, GIPC1 also links MYO6 to; a quadripartite complex containing LARG and SH3BP4; a separate complex containing CARD10; and likely other network components such as GULP1, FAM160A1, FMNL3 or LZTS2 that require further analysis. This therefore highlights the remarkable multi‐functionality of MYO6 at endosomes in conjunction with GIPC1.

### The LIFT complex regulates MYO6‐driven actin reorganisation

We next investigated a potential role of the MYO6‐GIPC1‐LARG‐SH3BP4 complex in regulating actin filament organisation, as LARG is a GEF for RHO GTPases and SH3BP4 localised to actin structures including filopodia at the cell surface (Fig 3D). For this analysis, we used an engineered mutant of MYO6 (MYO6^+^), which moves in the opposite direction to the wild‐type protein 30. As previously shown, this mutant clusters early endosomes and induces actin reorganisation, leading to the formation of filopodia protruding from the cell surface, above a cortical cluster of endosomes, in a GIPC1‐dependent manner 30. Indeed, in mutant MYO6^+^‐expressing cells GIPC1, SH3BP4 and LARG are all recruited to MYO6^+^‐induced filopodia (Fig 3E). Furthermore, depletion of LARG and SH3BP4 using siRNA markedly reduced the number of MYO6^+^ cells that generated filopodia. By contrast, the knock‐downs had no effect on GFP‐MYO10‐induced filopodia formation (Figs 3F and EV2E and F). Taken together, these results indicate a role for LARG and SH3BP4 in MYO6^+^‐dependent actin reorganisation in the cell cortex.

### LPAR1‐LARG‐RHO‐dependent actin reorganisation controls endosome positioning

**Figure 4:**
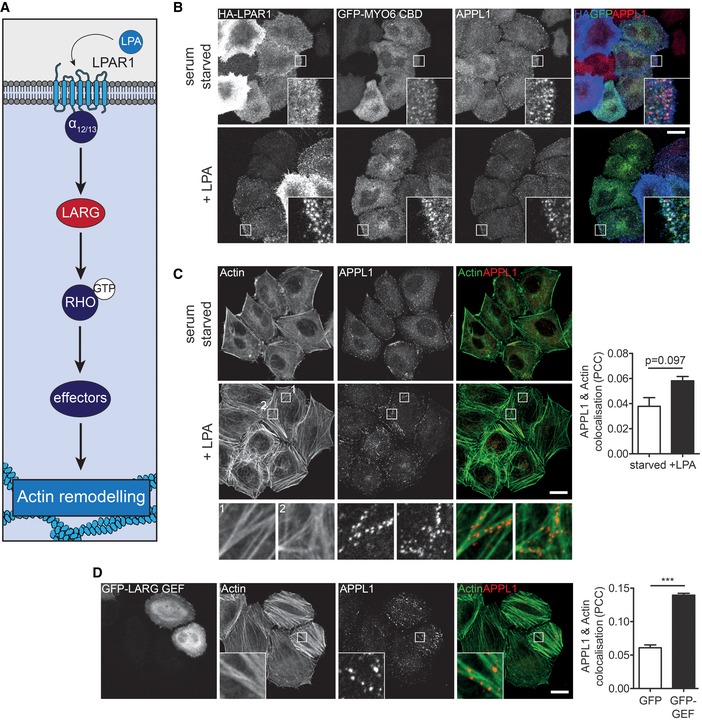
LPAR1‐LARG‐RHO‐dependent actin reorganisation controls endosome positioning Schematic of the LPA‐LARG‐RHO signalling pathway.Confocal microscope images of 0 min (upper panels) or 5 min (lower panels) LPAR1 uptake in HeLa cells expressing GFP‐MYO6 CBD and HA‐tagged LPAR1 in the presence of 10 μM LPA. Cells were immunostained for GFP (green), APPL1 (red) and LPAR1 (blue). Scale bar, 20 μm.Confocal microscope images of HeLa cells serum starved (upper panels) or treated with 10 μM LPA for 5 mins (lower panels) and immunostained for APPL1 (red) and actin (green). Scale bar, 20 μm. Graph to the right depicts the mean Pearson's correlation coefficient calculated for actin and APPL1 in serum starved or LPA stimulated cells from ≥ 5 fields of view (2–6 cells/field) in *n* = 3 independent experiments (> 70 cells per condition). Paired *t*‐test *P* = 0.097. Error bars indicate SEM.Confocal microscopy of HeLa cells expressing GFP‐LARG GEF immunostained for APPL1 (red) and actin (green). Scale bar, 20 μm. Graph to the right depicts the mean Pearson's correlation coefficient calculated for actin and APPL1 in GFP or GFP‐LARG GEF transfected cells from ≥ 7 fields of view (1–7 cells/field) in *n* = 4 independent experiments (> 100 cells per condition). Significance was calculated using a two‐sample *t*‐test. ***P < 0.001. Error bars indicate SEM.

**Figure EV3:**
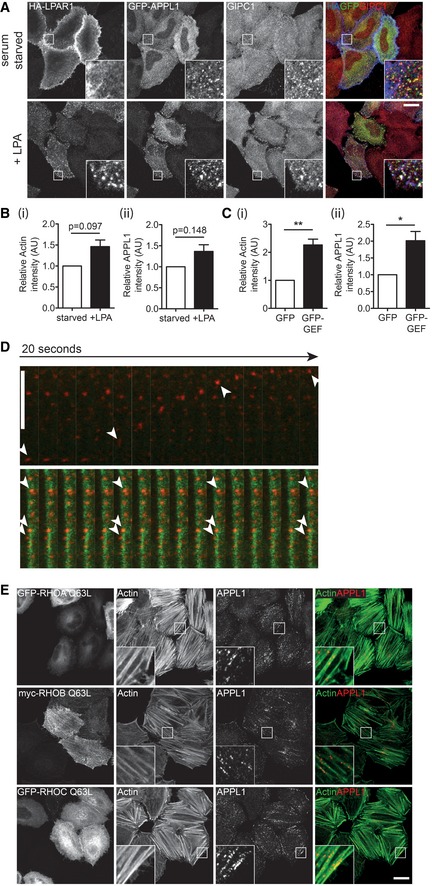
LPAR1‐LARG‐RHO‐dependent actin reorganisation controls endosome positioning and motility Confocal microscope images of HeLa cells expressing GFP‐APPL1 and HA‐tagged LPAR1 and serum starved overnight. Cell surface LPAR1 was labelled with HA antibody (blue), and uptake was allowed to proceed in the presence of 10 μM LPA for 0 min (upper panels) or 5 min (lower panels). Cells were fixed and immunostained with GFP (green) and GIPC1 (red) antibodies. Scale bar, 20 μm.Quantification of (i) phalloidin and (ii) APPL1 signal intensity in cells serum starved or treated with 10 μM LPA for 5 min. Data are the mean of *n* = 3 independent experiments. Paired *t*‐test *P* = 0.0974 (i) and *P* = 0.1481 (ii). Error bars indicate SEM.Quantification of (i) phalloidin and (ii) APPL1 signal intensity in GFP or GFP‐LARG GEF transfected cells. Graph depicts mean from *n* = 4 independent experiments. Significance was calculated using a one‐sample *t*‐test. *P < 0.05, **P < 0.01. Error bars indicate SEM.Image sequences from spinning disc confocal microscope showing mCherry‐APPL1 (red) and BFP‐LifeAct (green) in mock (top row) or GFP‐LARG GEF (bottom row) transfected HeLa cells. The motility of selected endosomes over time is highlighted by the arrowheads. Scale bar, 10 μm; 0.5 s/frame (see also Movie EV1).Confocal microscope images of HeLa cells transfected with GFP‐RHOA Q63L (top row), GFP‐RHOB Q63L (middle row) and GFP‐RHOC Q63L (bottom row). Cells were immunostained with an APPL1 antibody (red) and labelled with phalloidin to visualise actin (green). Scale bar, 20 μm.

LARG contains a regulator of G protein signalling (RGS) domain that can directly bind to the activated subunit of heterotrimeric G proteins such as Gα_12_ and _13_. Interestingly, LARG has previously been linked to signalling from GPCRs such as the lysophosphatidic acid receptor (LPAR1), that is also trafficked by GIPC1 and MYO6 through APPL1 endosomes (Figs 4A and B, and EV3A) 31, 32, 33. We therefore determined whether the MYO6‐GIPC1‐LARG‐SH3BP4 complex links LARG and RHO‐dependent actin organisation to GIPC1 endosome function and position. We analysed actin filament distribution and APPL1‐positive endosome localisation upon activation of the canonical LPAR1‐LARG‐RHOA signalling pathway after treating cells with LPA for 5 min. Quantitation revealed a modest non‐significant increase in actin filaments and APPL1 signal intensity as well as colocalisation of APPL1 with actin upon LPA stimulation (Figs 4C and EV3B). Likewise, overexpression of the unregulated LARG RhoGEF domain led to significant increases in actin bundles, APPL1 signal intensity and APPL1 colocalisation with actin, indicated by the dramatic alignment of APPL1‐positive endosomes along filaments (Figs 4D and EV3C). Live cell microscopy indicated that the APPL1 endosomes aligned along actin filaments had greatly reduced motility (Fig EV3D and Movie EV1). Finally, ectopic expression of constitutively active RHOA, RHOB and RHOC also triggered an increase in actin filament bundles and subsequent endosome recruitment (Fig EV3E).

Taken together, these data suggest that the LIFT complex is an actin regulatory module, which may function downstream of GPCRs such as LPAR1 to drive RHO‐mediated actin reorganisation to regulate endosome positioning and motility. These results support, and may provide the molecular mechanism for, our recent finding that MYO6 mediates association of APPL1 endosomes with cortical actin filaments 34, 30. Depletion of MYO6 or expression of the reverse MYO6^+^ affects endosome localisation in the cell cortex. In this way, MYO6 could either regulate endosome position directly through organelle tethering to actin filaments or indirectly through reorganisation of the actin cytoskeleton involving recruitment of RhoGEFs such as LARG.

### MYO6 is linked to the RhoGEF DOCK7 via LRCH3

**Figure 5:**
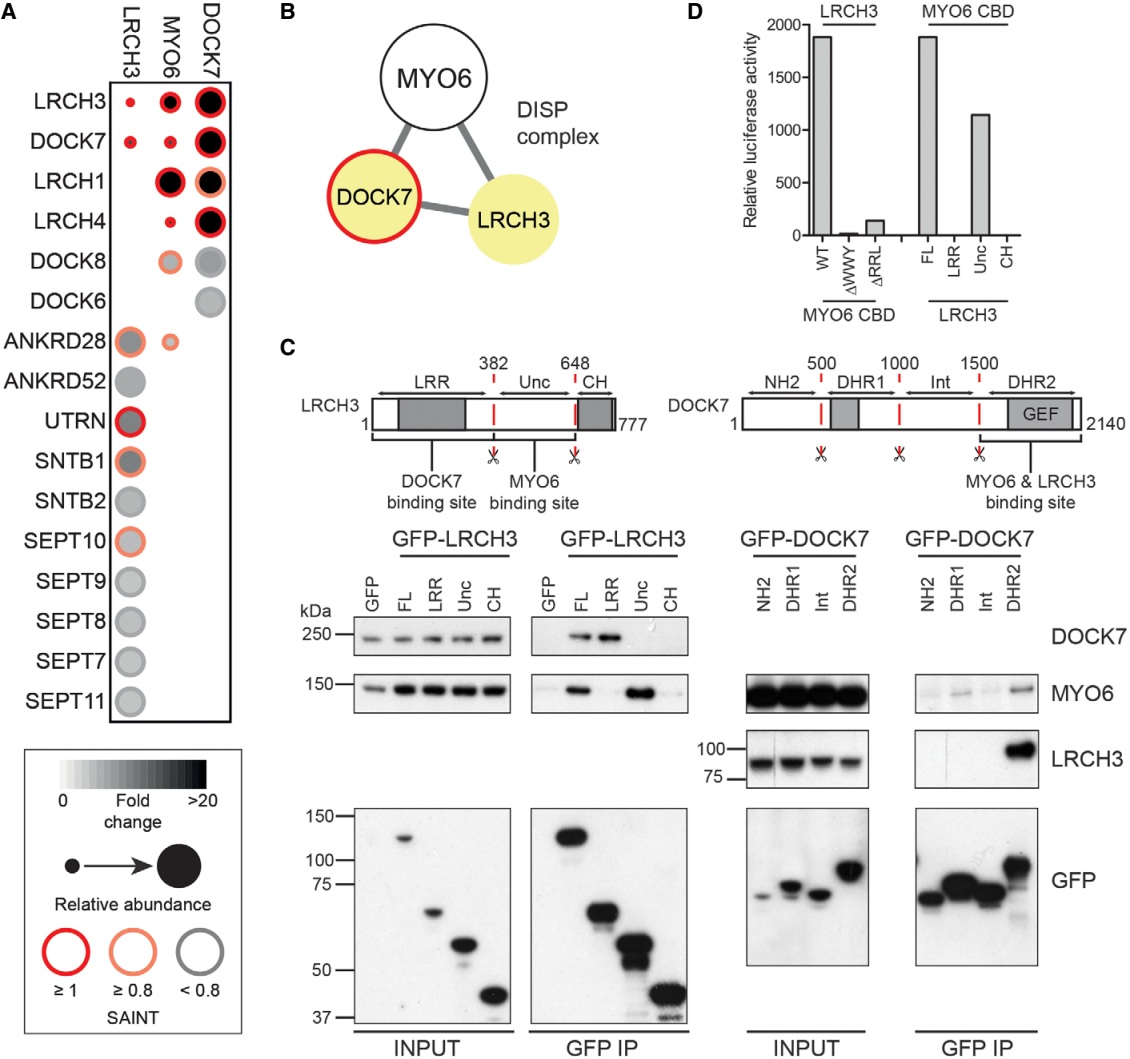
MYO6 is linked to the RhoGEF DOCK7 via LRCH3 Dot plot of high and medium confidence interactions (> 3 FC‐A and > 0.8 SAINT or > 3 FC‐A and < 0.8 SAINT) identified in BirA*‐LRCH3 and BirA*‐DOCK7 experiments and shared interactors from the BirA*‐MYO6 CBD interactome.Network diagram of the DISP complex.Top: Schematic cartoon highlighting domain structure, fragments and binding sites found in LRCH3 (left) and DOCK7 (right). Bottom: GFP nanobody immunoprecipitates from HEK293T cells transfected with GFP, full‐length GFP‐LRCH3 and GFP‐LRCH3 fragments corresponding to amino acids 1–382 (LRR), 383–648 (Unc) or 649–777 (CH) or GFP‐DOCK7 fragments corresponding to amino acids 1–500 (NH2), 501–1000 (DHR1), 1001–1500 (Int) or 1501–2140 (DHR2). Samples were analysed by Western blot with the indicated antibodies.The mammalian two‐hybrid assay was used to test direct binding of full‐length LRCH3 and wild‐type, ΔWWY or ΔRRL MYO6 tail and full‐length LRCH3 or LRCH3 fragments and wild‐type MYO6 tail. Graph shows relative luciferase activity from a single representative experiment. Source data are available online for this figure.

Our *in situ* proximity labelling and mutational profiling resolved another putative MYO6‐associated protein complex composed of DOCK7 and LRCH family proteins. DOCK7 is a GEF for RAC1 and CDC42, which forms a complex with MYO6 to regulate neurite outgrowth 35, 36. Secondary screens in BirA*‐LRCH3 expressing cells identified five high‐confidence interactions, as well as several lower confidence hits, including DOCK7, a number of septin family members, protein phosphatase 6 (PP6) components as well as utrophin and syntrophins (Figs 2 and 5A). BirA*‐DOCK7 labelling identified only three high‐confidence interactions, all of which were LRCH family proteins (LRCH1, LRCH3 and LRCH4), as well as lower confidence interactions with other DOCK proteins (Figs 2 and 5A). The LRCH3, DOCK7 and MYO6 data sets showed overlap with multiple LRCH and DOCK family proteins in addition to the PP6 component ANKRD28, indicating that LRCH3, DOCK7 and LRCH1 indeed form a distinct MYO6‐associated protein complex (Fig 5B).

We next confirmed the interactions between MYO6, LRCH3 and DOCK7 and mapped the topology of the complex by performing pull‐down experiments using full‐length and functional domain fragments of LRCH3 and DOCK7. These experiments showed both MYO6 and DOCK7 can bind to LRCH3; DOCK7 binds to its leucine‐rich repeats and MYO6 to a region between amino acids 383–648 (Fig 5C). Analysis of IPs with different GFP‐DOCK7 fragments indicated that both MYO6 and LRCH3 bound to the DHR2 domain of DOCK7 (Fig 5C). The observations that MYO6 and LRCH3 both bound to the same site on DOCK7, but MYO6 and DOCK7 bound distinct sites on LRCH3 imply that LRCH3 is the linker between MYO6 and DOCK7. Indeed, our M2H assay confirmed that LRCH3 interacts directly with MYO6; the binding site again mapped to amino acids 363–648 and required both the WWY and RRL motif in the MYO6 CBD, corroborating our earlier SILAC experiments (Figs 1E and 5D).

**Figure EV4:**
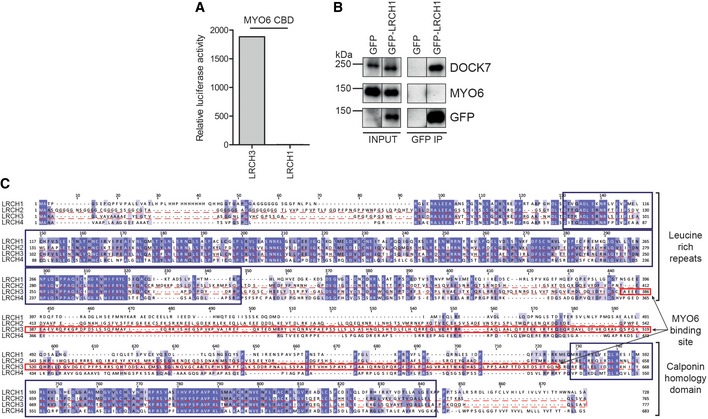
LRCH1 is not a MYO6 binding partner The mammalian two‐hybrid assay was used to test binding of full‐length LRCH3 and LRCH1 to wild‐type MYO6 tail. Graph shows the mean relative luciferase activity from a single representative experiment.GFP nanobody immunoprecipitates from HEK293T cells transfected with GFP and full‐length GFP‐LRCH1 were analysed by Western blot with the indicated antibodies (GFP control IP same as Fig 5C).Sequence alignment of the LRCH proteins. Boxes highlight the highly conserved leucine‐rich repeats and calponin homology domains (blue) and the unconserved MYO6 binding site in LRCH3 (red).

Although other LRCH family members such as LRCH1 were identified in the MYO6 and DOCK7 BioID data sets, no interaction between LRCH1 and MYO6 was observed, although LRCH1 did co‐immunoprecipitate DOCK7 (Fig EV4A and B). Indeed, LRCH family proteins show high levels of conservation within the N‐terminal LRRs at the site of DOCK7 binding, but very little in the region of the MYO6 binding site (Fig EV4C).

### The DISP complex regulates septin organisation

**Figure EV5:**
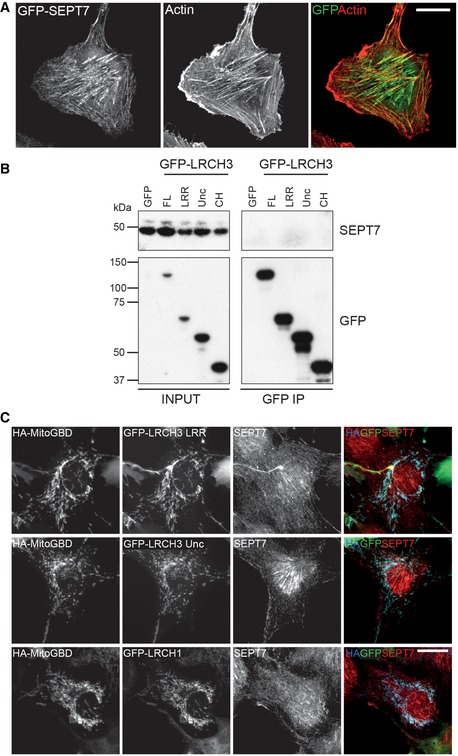
The DISP complex regulates septin organisation Confocal microscope images of RPE cells stably expressing GFP‐SEPT7 and untransfected and immunostained with a GFP (green) antibody and labelled with phalloidin to visualise actin (red). Scale bar, 20 μm.GFP nanobody immunoprecipitates from HEK293T cells transfected with GFP, full‐length GFP‐LRCH3 and GFP‐LRCH3 fragments corresponding to amino acids 1–382 (LRR), 383–648 (Unc) or 649–777 (CH). Samples were analysed by Western blot with the indicated antibodies (same IP as Fig 5C).Widefield microscope images of RPE cells transfected with HA‐MitoGBD and GFP‐LRCH3 fragments corresponding to amino acids 1–382 (LRR; top row) and 383–648 (Unc; middle row) or full‐length GFP‐LRCH1 (bottom row). Cells were immunostained with HA (blue), GFP (green) and SEPT7 (red) antibodies. Scale bar, 20 μm.

**Figure 6:**
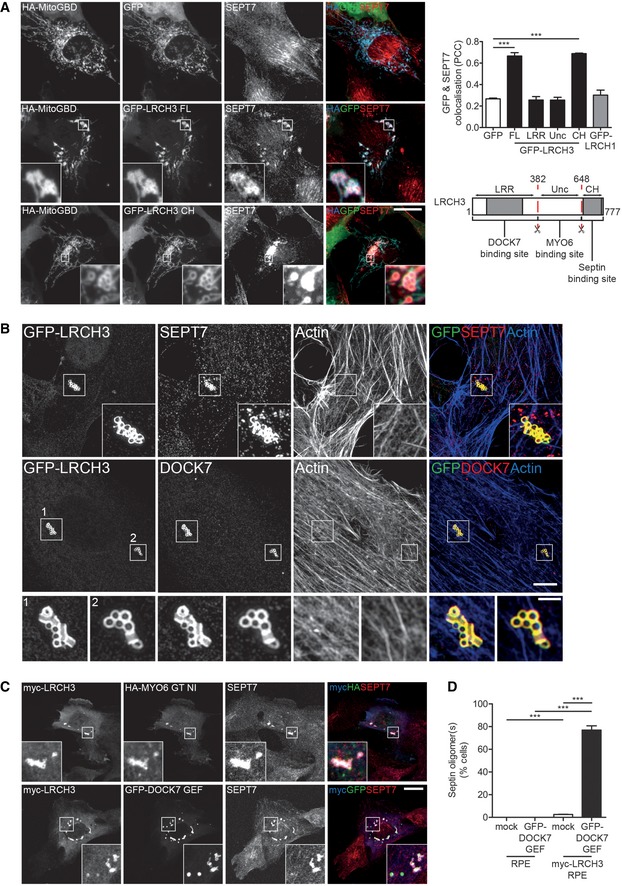
The DISP complex regulates septin organisation Widefield microscope images of RPE cells expressing HA‐MitoGBD and GFP (top row), full‐length GFP‐LRCH3 (middle row) or GFP‐LRCH3 fragment 649–777 (CH; bottom row). Cells were immunostained with HA (blue), GFP (green) and SEPT7 (red) antibodies. Scale bar, 20 μm. Graph to the right depicts the Pearson's correlation coefficient calculated for GFP and SEPT7 in RPE cells transfected with GFP, full‐length GFP‐LRCH3, GFP‐LRCH3 fragments and GFP‐LRCH1. Graph displays the mean calculated from 10 fields of view (1 cell/field) from *n* = 3 independent experiments. Statistical significance was determined by repeated measures ANOVA and a Bonferroni post hoc test. ***P < 0.001. Error bars indicate SEM. Below, schematic cartoon highlighting domain structure of LRCH3 and the putative SEPT7 binding site.Structured illumination microscope images of RPE cells transiently transfected with GFP‐tagged LRCH3, immunostained with GFP (green), SEPT7 (top row) or DOCK7 (bottom row, red) antibodies and labelled with phalloidin to visualise actin (blue). Scale bar, 5 μm or inset, 1 μm.Confocal microscope images of RPE cells stably expressing myc‐LRCH3 and transiently transfected with HA‐MYO6 CBD NI (upper panels) or GFP‐DOCK7 DHR2 domain (lower panels, GFP‐DOCK7 GEF). Cells were immunostained with myc (blue), HA (top row, green) or GFP (bottom row, green) and SEPT7 (red) antibodies. Scale bar, 20 μm.Graph depicting the mean percentage of GFP‐DOCK7 GEF or myc‐LRCH3‐positive cells which displayed septin oligomerisation. Counts were performed on > 100 cells per condition from *n* = 3 independent experiments. Statistical significance was determined by two‐sample *t*‐test. ***P < 0.001. Error bars indicate SEM.

Finally, our BioID experiments identified a possible interaction between LRCH3 and a number of septins. Septins are a family of GTPases, which oligomerise to form filaments and other higher order structures, templated by the actin cytoskeleton 37. At steady state, septins localise along actin bundles in the cell body, but are absent from more dynamic membrane ruffles (Fig EV5A). As we were unable to observe septins in LRCH3 IPs (the same IP was positive for MYO6 and DOCK7; compare Figs 5C and EV5B), we verified this interaction using a knock sideways approach that targeted GFP‐LRCH3 to mitochondria using a GFP binding domain fused to a mitochondria‐targeting sequence (MitoGBD). Co‐expression of MitoGBD with GFP alone, full‐length GFP‐LRCH3 or GFP‐tagged domain fragments of LRCH3 in RPE cells caused all to relocalise to the mitochondria; however, only relocalisation of full‐length LRCH3 or a fragment containing its calponin homology domain triggered the concomitant recruitment, and oligomerisation, of SEPT7, from its steady state localisation along actin filaments to mitochondria (Figs 6A and EV5C). Interestingly, despite sharing a highly conserved calponin homology domain with LRCH3, full‐length GFP‐LRCH1, which does not interact with MYO6, failed to relocalise septins (Figs 6A and EV5C). Overexpression of LRCH3 alone does not lead to any obvious changes in actin filament organisation but led to the displacement of septins from actin filaments and the assembly of cytosolic ring‐like septin structures in a small but significant population of cells (Fig 6B–D). To determine whether MYO6 and DOCK7 also localised to these septin structures, we co‐expressed HA‐MYO6 CBD or GFP‐DOCK7 DHR2 and found both co‐localised with myc‐LRCH3 and SEPT7 (Fig 6B and C). Strikingly however, the co‐expression of LRCH3 and the DOCK7 DHR2 domain, which contains the LRCH3 and MYO6 binding sites as well as the RAC1/CDC42 GEF activity of DOCK7, caused a very dramatic increase in the number of cells (~80%) containing septin ring structures (Fig 6D). These data provide the first evidence for a possible role of LRCH3 and DOCK7 in the remodelling of the septin cytoskeleton.

## Discussion

In this study, we have used proximity labelling‐based proteomics to follow the dynamic interactions of the cargo‐binding tail of MYO6 *in situ*. To our knowledge, this is the first *in vivo* interaction map identified for a myosin motor protein. Using proximity‐dependent labelling in the native cellular environment, we have successfully identified and, by comprehensive biochemical characterisation, validated a number of novel protein complexes that link MYO6 to hitherto unknown functions. The MYO6 interactome contains over 100 proteins which we have grouped into several distinct functional complexes. This large network of interactions is likely to be required for the multitude of cellular tasks that require this reverse actin‐based motor in mammalian cells.

**Figure 7:**
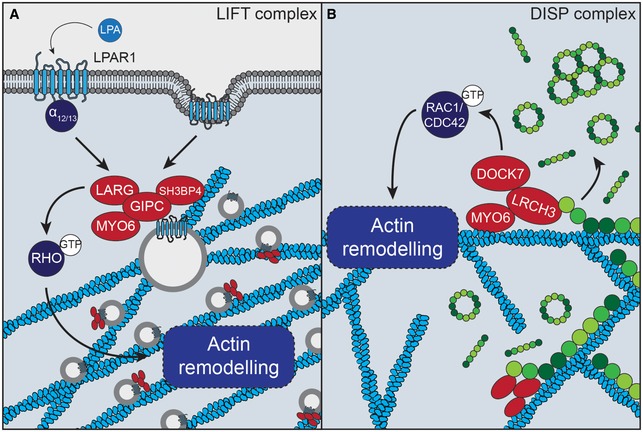
Model of LIFT/DISP complex function Upon activation of Gα12/13‐coupled receptor at the cell surface (e.g. LPAR1), LARG is activated and the receptor is internalised into MYO6‐GIPC1‐positive endosomes. LARG is able to catalyse the GDP‐GTP exchange of RHO GTPases which can then activate their downstream effectors to promote actin remodelling. The interaction between LARG and GIPC1 links this activity to the endosome to promote actin reorganisation in proximity to the trafficking receptor. This actin remodelling might affect endosome position and motility.LRCH3 is able to displace septins (green) from actin filaments (blue) via its C‐terminal calponin homology domain. Once septins are displaced from the actin, DOCK7 can promote actin remodelling via its activity towards RAC1 and CDC42 GTPases.

Most notably, we have identified two new MYO6 complexes containing RhoGEFs, which regulate the actin cytoskeleton and thereby receptor trafficking at early endosomes or modulation of the septin cytoskeleton. The first is LARG, a GEF for RHO GTPases, which is one of the 15 shared interactions between MYO6 and GIPC1. Together with SH3BP4, these three proteins exist in a quadripartite complex, the LIFT (LARG‐Induced F‐actin for Tethering) complex, which we implicate in actin modulation at early endosomes. We show that the GEF activity of LARG or LPAR1‐LARG‐RHO signalling can affect the positioning and motility of MYO6‐GIPC1‐positive endosomes. The role of MYO6 and GIPC1 in the transport of vesicles and receptors through the cortical actin network is well established 38, 39, 40; thus, by linking MYO6‐GIPC1 to LARG, we highlight the possibility that LARG and SH3BP4 might be recruited to endosomes to trigger localised LARG‐RHO‐driven remodelling of the actin cortex to modulate vesicle trafficking. The perturbation in filopodia formation by engineered mutant MYO6^+^ upon depletion of either LARG or SH3BP4 would seem to support this. Furthermore, as LARG is activated by the stimulation of specific GPCRs (e.g. LPAR1) which traffic through MYO6‐GIPC1 endosomes 31, 32, 33, it is attractive to hypothesise that the interaction between LARG and GIPC1 might serve as a feed‐forward mechanism to regulate the subsequent retrograde movement of internalised receptors (Fig 7A). In addition, these endosomes are thought to serve as signalling platforms. As such, this ability to modulate vesicle motility is likely to mediate not only receptor traffic and degradation but also the duration of signal transduction. Indeed, we have recently shown a role for MYO6 and GIPC1 in regulating Akt signalling on this compartment 34 and it will be interesting to further address the role of the LIFT complex in this pathway.

The second RhoGEF, DOCK7, is a GEF for both RAC1 and CDC42 41 and has previously been associated with MYO6 35, 36. Here, we show DOCK7 links via LRCH3 to MYO6 to form the septin regulatory DISP (DOCK7‐Induced Septin disPlacement) complex. Interestingly, proximity‐dependent labelling with LRCH3 identified a link to septins, important components of the cytoskeleton. In mammalian cells, septins can assemble into higher order structures including rings and filaments. These filaments have been linked to the actin cytoskeleton by colocalisation and observations that perturbation of the actin cytoskeleton causes the formation of septin rings or, conversely, septin depletion triggers the loss of actin 37, 42, 43. Our experiments show overexpression of the DISP complex promotes the formation of septin rings, indicative of septin displacement from actin, demonstrating that these proteins are a novel class of septin regulator. Interestingly, we and others observe that septins filaments are largely excluded from dynamic actin structures such as membrane ruffles and appear to stabilise actin bundles 42, 43. RAC1 and CDC42 are both potent regulators of actin dynamics and septin organisation 43, 44, and it is therefore tempting speculate that DOCK7 might exert its effect on the septin cytoskeleton via its GEF activity towards those GTPases (Fig 7B). Further work will be required to determine the precise mechanistic details of this, but we propose that the DISP complex might locally coordinate removal of the actin stabilising septin scaffold to allow MYO6‐dependent actin remodelling driven by DOCK7.

Together, this work highlights an emerging paradigm in myosin function, the coordination of myosin activity and actin filament assembly. Specifically, class IX myosins have RhoGAP domains in their tails, MYO5 has recently been shown to coordinate motor targeting with assembly of actin tracks by binding to the actin nucleator SPIRE2 19, and myosins of class I can interact directly with proteins that regulate actin patch formation at the plasma membrane 20, 21, 22. Our identification of two distinct MYO6‐associated RhoGEF complexes, which link to different actin modulatory pathways, builds significantly on this regulatory theme and highlights the extent to which motor activity is synchronised with actin track assembly. Overall our results suggest that myosin motors do not simply translocate along pre‐existing actin filaments, but can actively induce actin tracks as required.

Advanced proteomics provides an exciting opportunity to explore the regulation and function of transient complexes formed by motor proteins. Although some known partners such as the autophagy receptors NDP52, TAX1BP1 or OPTN were not identified in our BioID experiments, this likely reflects their MYO6 interactions occur in a tissue‐specific or temporally‐restricted manner. Additional proteomics studies could be adapted to uncover such complexes. Nonetheless, the identification of > 90 novel MYO6 interacting proteins and two new regulatory complexes highlights the power of this technology and provides a rich resource for the cytoskeletal community. With advances in proteomic analyses of post‐translational modifications such as phosphorylation and ubiquitination, this approach could have further utility in determining how specific signalling pathways regulate myosin motor activity and cargo binding in specific subcellular contexts.

## Materials and Methods

### Antibodies and reagents

Antibodies used in this work were as follows: myc (05‐724; 1:200 IF, 1:2,000 WB) monoclonal antibody (Millipore); DAB2 (sc‐13982; 1:200 IF, 1:2,000 WB), APPL1 (sc‐67402; 1:100 IF, 1:1,000 WB), SH3BP4 (sc‐393730; 1:50 IF, 1:500 WB), LARG (sc‐25638; 1:50 IF), SEPT7 (sc‐20620, 1:50 IF, 1:1,000 WB) and EF2 (sc‐13004; 1:2,000 WB) antibodies (Santa Cruz); GIPC1 (25‐6792; 1:100 IF, 1:1,000 WB) polyclonal antibody (Proteus); GAPDH (G8795; 1:10,000 WB) monoclonal antibody (Sigma); HA (11867423001, 1:200 IF) antibody (Roche); GFP (A11122; 1:400 IF) polyclonal antibody (Life Technologies); GFP (ab1218; 1:400 IF) monoclonal antibody (Abcam); affinity‐purified rabbit polyclonal antibodies against GFP (1:2,000 WB) and MYO6 (1:100 IF, 1:1,000 WB) were generated as described previously 45. Affinity‐purified rabbit polyclonal antibodies against amino acids 383–648 of LRCH3 (1:1,000 WB) or amino acids 1500–2140 of DOCK7 (1:1,000 WB) were generated as part of this work.

### Plasmids

The pcDNA3.1 myc‐BirA (R118G) generated by Kyle Roux was obtained from Addgene (35700 23) and subcloned into the pLXIN retroviral vector. MYO6 CBD LI (isoform 1, Q9UM54‐1, amino acids 1037–1285) and MYO6 CBD NI (isoform 5, Q9UM54‐5, amino acids 1036–1253) WT, ΔWWY, ΔRRL and ΔPIP_2_ were amplified by PCR from MYO6 FL and tail wild‐type, ΔWWY, ΔRRL and ΔPIP_2_ pEGFPC constructs described elsewhere 8, 46, 47, 48 and inserted in‐frame at the 3′ end of the BirA* tag. Mammalian two‐hybrid MYO6 tail wild‐type and mutant pM constructs have been described elsewhere 9, as have GFP‐MYO6^+^ and GFP‐MYO10 30. HA‐MYO6 CBD was generated by PCR using primers containing the HA tag sequence.

Full‐length GIPC1 (isoform 1, UniProtKB O14908‐1) and DOCK7 (isoform 4, Q96N67‐4) were generated by PCR from RPE cDNA. Full‐length LARG, SH3BP4, LRCH3 and LRCH1 cDNA (clone IDs: 184137, 6138465, 2960711 and 40126023, respectively) were obtained from Thermo Fisher Scientific, and CARD10 cDNA (clone ID: 8322711) was obtained from GE Healthcare. Full‐length inserts or truncations were amplified by PCR and ligated into pEGFPC, pVP16, pM or myc‐BirA* pLXIN2 as relevant. To generate myc‐LRCH3 RPE stable cell lines, full‐length LRCH3 was ligated into pCMV‐myc and subcloned into pLXIN retaining the myc tag.

GFP‐CDC42 (12600) and GFP‐RAC1 (13720) generated by Klaus Hahn; GFP‐RHOA Q63L (12968) by Gary Bokoch; and RHOC pEGPFC2 (23226) and RHOD pcDNA3 (23235) by Channing Der were all obtained from Addgene 49, 50, 51, 52. myc‐RHOB pcDNA was a kind gift from Philip Woodman (University of Manchester). RHOB, RHOC and RHOD were mutated by site‐directed mutagenesis to generate the corresponding constitutively active mutants. HA‐LPAR1 was obtained from the Harvard PlasmID repository (HsCD00000181) and subcloned into pIRES‐neo2. SEPT7 (isoform 1, Q16181‐1) was generated by PCR from RPE cDNA and ligated into pEGFPC or pIRES‐neo2 retaining the GFP tag. The pOPINE GFP nanobody (49172) plasmid generated by Brett Collins was obtained from Addgene 53. The HA‐MitoGBD pCAG was generated by PCR amplification of the GFP nanobody (GBD) insert and ligation into HA‐MitoFKBP pCAG, a kind gift from Margaret Robinson (University of Cambridge)

### Cell culture and transfections

HeLa cells were cultured in RPMI‐1640, CHO cells in F12 HAM and RPE cells in a 1:1 mixture of DMEM and F12 HAM. All medium contained sodium bicarbonate and was supplemented with 10% FBS, 2 mM l‐glutamine, 100 U/ml penicillin and 100 μg/ml streptomycin. HEK293T cells and the derivative Phoenix retrovirus producer cell line were cultured in DMEM containing GlutaMAX™ and supplemented with 10% FBS, 100 U/ml penicillin and 100 μg/ml streptomycin. For comparative proteomics experiments, cell populations were labelled with heavy or light amino acids. RPE cells were cultured through three passages in SILAC DMEM:F12 (1:1) medium (Thermo Fisher Scientific) supplemented with 10% dialysed FBS (Gibco) and 147.5 mg/l l‐arginine ^13^C_6_^15^N_4_ (Arg10; Cambridge Isotope Laboratories) and 91.25 mg/l l‐Lysine ^13^C_6_^15^N_2_ (Lys8; Cambridge Isotope Laboratories) heavy amino acids or their light equivalents.

DNA transfections were performed using FuGENE^®^6 transfection reagent (Promega) according to the manufacturer's instructions for both RPE and HeLa cells, or using PEI (Polysciences) for HEK293T cells. For RNAi‐mediated gene silencing siRNA ON‐TARGETplus SMARTpool oligonucleotides (Dharmacon, GE Healthcare) targeting LARG and SH3BP4 were transfected into cells using Oligofectamine™ (Invitrogen) according to the manufacturer's instructions. To ensure optimal knock‐down, all cells were transfected on day 1 and again on day 3, seeded according to application on day 4 and assayed on day 5.

For BioID experiments, cells were treated with complete medium supplemented 50 μM biotin (Sigma) for 24 h. For LPA stimulation experiments, HeLa cells were starved overnight in serum‐free RPMI and stimulated with 10 μM LPA (Sigma) for the indicated time.

### Generation of stable cell lines

RPE cell lines stably overexpressing myc‐BirA* fusion proteins or myc‐LRCH3 were generated by retroviral transduction. Virus was generated by transfecting the Phoenix retroviral producer line with the pLXIN retroviral packaging vector. After 48 h, virus was harvested and used to infect parental RPE cells before selection with 500 μg/ml G418 (Gibco). RPE cells stably expressing GFP‐SEPT7 were generated by stable transfection and selection in G418. GFP‐positive populations were subsequently enriched by fluorescence‐activated cell sorting.

### Immunofluorescence microscopy

Cells were grown on sterilised coverslips, fixed in 4% formaldehyde, permeabilised with 0.2% Triton X‐100 and blocked with 1% BSA in PBS before incubation with the relevant primary antibody and, subsequently, with AlexaFluor^®^488/568/647‐conjugated secondary antibodies (Molecular probes). DNA was visualised with Hoechst, biotin with AlexaFluor^®^568‐conjugated streptavidin (Molecular probes) and actin with AlexaFluor^®^488/568/647‐conjugated phalloidin (Molecular probes). For structured illumination microscopy experiments, cells were grown on acid‐washed, No. 1.5, 18 mm square coverslips (high performance 170 ± 5 μm, Schott, Germany). For antibody uptake experiments, cells were starved overnight, incubated for 15 min at 4°C with HA antibody before uptake at 37°C in the presence of 10 μM LPA for the indicated time. Images were obtained using a 63× objective on a Zeiss LSM710 confocal microscope, a Zeiss AxioImager upright widefield epifluorescence microscope equipped with an ORCA Flash 4 v2 camera or, for SIM, on a Zeiss Elyra PS1 super‐resolution microscope. To measure colocalisation, images were taken from randomly selected fields of view, background subtracted and cells manually segmented using ImageJ. The Pearson's correlation coefficient was then calculated using the ImageJ plugin coloc2. Statistical analysis was performed in GraphPad Prism as indicated.

### Mammalian two‐hybrid assay

To map direct interactions, bait and prey were amplified by PCR and ligated into the pVP16 or pM vectors (Clontech). Mammalian two‐hybrid experiments were performed in CHO cells as described previously 54.

### Nanobody purification and Affi‐Gel conjugation

GBD pOPINE was expressed in C41 *E. coli* cells and purified as described in 14, 45. GFP nanobody was coupled to Affi‐Gel 10 resin (Bio‐Rad) as per the manufacturer's instructions.

### Western blotting and immunoprecipitation

Cells lysates were prepared in 1% NP‐40 buffer (50 mM Tris–HCl at pH 7.5, 150 mM NaCl, 1 mM EDTA, 1% NP‐40 and complete protease inhibitor cocktail [Roche]) on ice, separated by SDS–PAGE and transferred to Immobilon‐FL polyvinylidene difluoride (PVDF) membrane (Millipore) by wet transfer. Membranes were blocked in 5% milk, incubated overnight at 4°C with primary antibodies diluted in 5% milk and HRP‐conjugated secondary antibodies (α‐rabbit/mouse/goat IgG‐HRP, Sigma) in 5% milk for 1 h at room temperature; 5% BSA was used to block streptavidin‐HRP blots. Membranes were developed using enhanced chemiluminescence (ECL) substrate (GE Healthcare) and exposure to Super RX‐N medical X‐ray film (Fuji).

Immunoprecipitations were performed with HEK293T cells growing on 100‐mm dishes. 24 h post‐transfection cells were lysed with 1% NP‐40 lysis buffer, homogenised using a 25G needle and clarified by centrifugation at 20,000 × *g* for 15 min at 4°C. Lysates were precleared with TBS‐blocked Affi‐Gel resin before incubating for 3 h with a 10 μl bead bed of GFP‐nanobody Affi‐gel resin. After washing with lysis buffer three times and TBS twice, proteins were eluted using SDS sample loading buffer and boiling before analysis by immunoblot.

### BioID purification and sample processing

For large‐scale MS BioID experiments, cells were seeded onto 2× 150‐mm dishes. At 50% confluency, cells were fed with fresh complete growth medium supplemented with 50 μM biotin and incubated for 24 h to allow labelling. Biotinylated cells were lysed with RIPA lysis buffer (50 mM Tris–HCl [pH 7.5], 150 mM NaCl, 1% NP‐40, 0.5% sodium deoxycholate, 1 mM EDTA, 0.1% SDS and complete protease inhibitor cocktail), homogenised using a 25G needle and, after sonification, clarified by centrifugation. Clarified lysates were mixed with high capacity streptavidin beads (Thermo Scientific, #20357) for 3 h at 4°C. Beads were washed with RIPA buffer three times, TBS twice and ammonium bicarbonate pH 8 (ABC, Sigma) before incubation for 30 min at 56°C in 10 mM DTT (Sigma, BioXtra). The solution was spiked with 10 μl 550 mM Iodoacetamide (IAA, Sigma, BioUltra) incubated for 20 min and washed before digestion overnight in 50 mM ABC containing 0.5 μg of Trypsin Gold (Promega). An additional 0.5 μg of trypsin was added the following day and incubated for a further 2 h at 37°C. The supernatant was collected, and beads were washed twice with 150 μl of HPLC‐grade H_2_O (Sigma, CHROMASOLV^®^) and all supernatants combined. The pooled eluant was spiked with 1 μl of 100% trifluoroacetic acid (TFA) and dried to a pellet in a vacuum centrifuge. For SILAC BioID experiments, heavy and light amino acid‐labelled cells were each seeded onto individual 150‐mm dishes. Lysates were quantified using the Precision Red Advanced Protein Assay Kit (Cytoskeleton) as per the manufacturer's instructions and equal amounts of protein pooled before processing in the same way.

### MS acquisition and data analysis

Samples were resuspended in MS solvent (3% acetonitrile, 0.1% TFA) for analysis on a Q Exactive (Thermo Scientific) coupled to an RSLC3000nano UPLC (Thermo Scientific). Peptides were resolved using a 50 cm C18 PepMap EASYspray column with a gradient rising from 97% solvent A (0.1% formic acid), 3% solvent B (80% acetonitrile, 0.1% formic acid) to 40% solvent B over 40 min. Data were acquired in a top 10 data‐dependent acquisition fashion with MS spectra acquired between *m/z* 400 and 1,400 at 70,000 fwhm. MS‐MS spectra were acquired at 17,500 fwhm and excluded from further fragmentation for 30 s.

Raw files were processed as a single batch using the MaxQuant proteomics software package version 1.5.0.0 55. Spectra were searched using the built‐in Andromeda search engine and the UniProt reference database for human proteins. Cysteine carbamidomethylation was set as a fixed modification, and methionine oxidation and N‐terminal acetylation were selected as variable modifications. Both peptide and protein false discovery rates (FDRs) were set to 0.01, the minimum peptide length was set at seven amino acids, and up to two missed cleavages were tolerated. Some bioinformatics analysis was performed in the Perseus package bundled with MaxQuant 56. Data were filtered by removing matches to the reverse database, proteins only identified with modified peptides, and common contaminants and intensity values were log_10_ transformed. For label‐free experiments, data were uploaded to the CRAPome.org online analysis tool 25, 26. The default settings were used for all analysis in CRAPome (FC‐A, user, default, average; FC‐B, all, stringent, geometric; SAINT‐express, user, average, virtual controls 10, all replicates). Scores were downloaded and exported to ProHits‐Viz to make dot plots or to Cytoscape for network diagrams 57, 58.

For SILAC experiments, heavy/light ratios were log_2_ transformed and outliers were identified using the significance A function (Benjamini–Hochberg procedure) in Perseus defining a threshold of 0.05. The significance A function was run on each triplicate ΔWWY, ΔRRL and ΔPIP2 experiment and gene names and SILAC ratios were compiled and carried forward for subsequent analysis. Principal component analysis (PCA) was performed in R using the mean heavy/light ratios of each significant protein and the “prcomp” and “biplot” functions.

The mass spectrometry proteomics data have been deposited to the ProteomeXchange Consortium via the PRIDE 59 partner repository with the data set identifier PXD008686.

## Author contributions

TO designed and performed experiments, analysed the data and wrote the manuscript. TAM generated Fig 3F and FB conceived the study, discussed the experiments and wrote the manuscript.

## Conflict of interest

The authors declare that they have no conflict of interest.

## Supporting information



Expanded View Figures PDF

Movie EV1

Review Process File

Source Data for Figure 5
