# Supplementary material for: The MYO6 interactome reveals adaptor complexes coordinating early endosome and cytoskeletal dynamics
Source: EMBO Rep. 2018 Feb 21;19(4):e44884. doi: 10.15252/embr.201744884 (PMC5891429; doi:10.15252/embr.201744884)
Supplement: Supplementary file 1 — Expanded View Figures PDF [file EMBR-19-e44884-s001.pdf]

Expanded View Figures

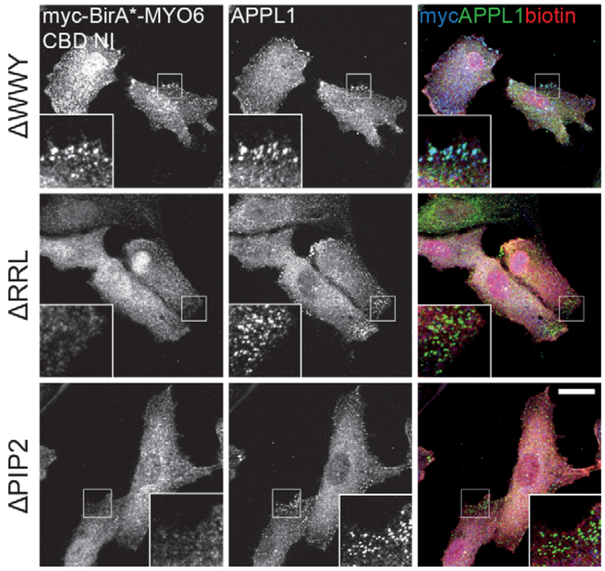

Figure EV1. Characterisation of BirA*-MYO6 CBD NI mutant cell lines.

Immunofluorescence microscope images of RPE cells stably expressing myc-BirA*-MYO6 CBD NI ΔWWY (top row), ΔRRL (middle row) and ΔPIP2 (bottom row) treated with 50 μM biotin for 24 h. Cells were immunostained with myc (blue) and APPL1 (green) antibodies. Scale bar, 20 μm.

Figure EV2. GIPC1 links MYO6 to multiple protein complexes.

A, B Top: The mammalian two-hybrid assay was used to test binding of (A) full-length GIPC1 against fragments of LARG encompassing amino acids 1–274 (PDZ-CC), 274–721 (RGSL), 721–1172 (DH-PH) and 1171–1544 (COOH) or LARG COOH against fragments GIPC1 encompassing amino acids 1–120 (NH2), 1–223 (NH2-PDZ), 120–223 (PDZ), 120–333 (PDZ-COOH), 223–333 (COOH) and (B) full-length SH3BP4 against fragments GIPC1 encompassing amino acids 1–120 (NH2), 120–223 (PDZ), 223–333 (COOH). Graphs show the mean relative luciferase activity from single representative experiments. Bottom: Schematic cartoon highlighting domain structure, fragments and binding sites found in LARG (left) and GIPC1 (right).

C GFP nanobody immunoprecipitates from HEK293T cells transfected with GFP and GFP-tagged CARD10 full length or missing the C-terminal four amino acids (ΔSEA). Samples were analysed by Western blot with the indicated antibodies.

D The mammalian two-hybrid assay was used to test binding of full-length CARD10 and CARD10 ΔSEA to full-length GIPC1. Graphs show the mean relative luciferase activity from single representative experiments.

E Widefield microscope images of HeLa cells transfected with GFP-MYO6^{+} or GFP-MYO10 and treated with mock, LARG or SH3BP4 siRNA. Cells were immunostained with a GFP antibody. Scale bar, 20 μm.

F HeLa cells treated with mock, LARG or SH3BP4 siRNA were analysed by Western blot using LARG, SH3BP4 and GAPDH (loading control) antibodies.

© 2018 The Authors

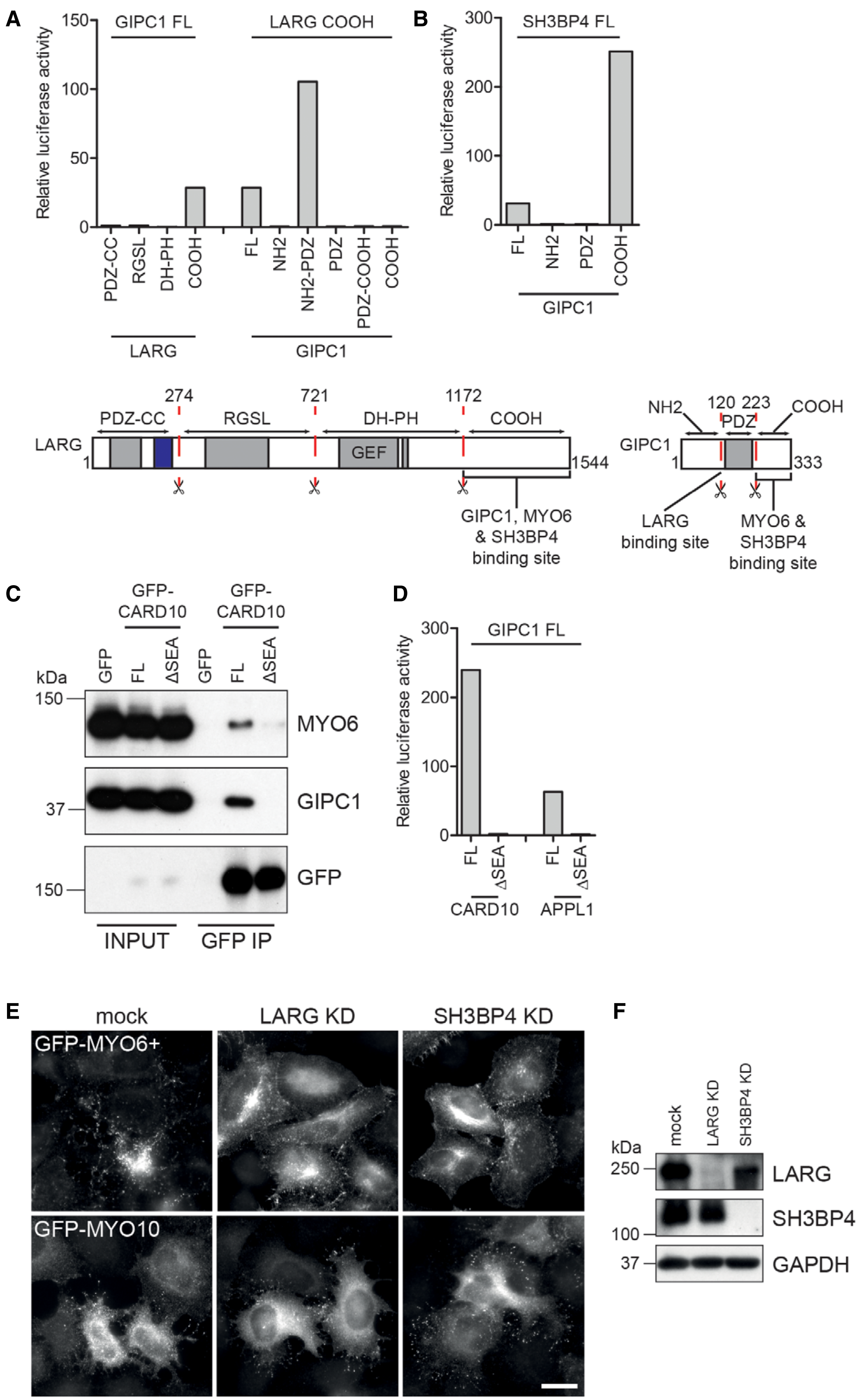

Figure EV2.

 © 2018 The Authors

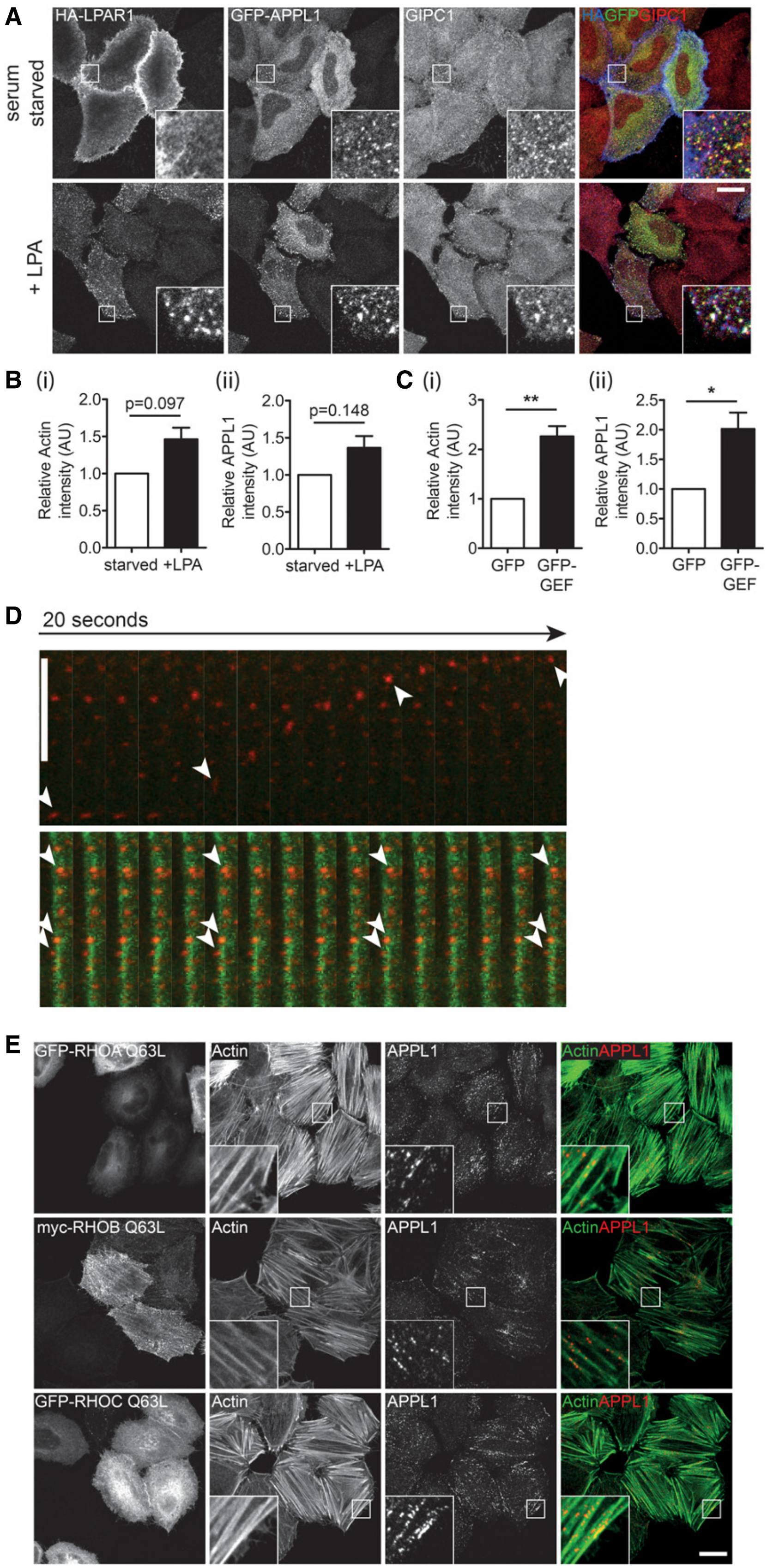

Figure EV3. LPAR1-LARG-RHO-dependent actin reorganisation controls endosome positioning and motility.

A Confocal microscope images of HeLa cells expressing GFP-APPL1 and HA-tagged LPAR1 and serum starved overnight. Cell surface LPAR1 was labelled with HA antibody (blue), and uptake was allowed to proceed in the presence of 10 μM LPA for 0 min (upper panels) or 5 min (lower panels). Cells were fixed and immunostained with GFP (green) and GIPC1 (red) antibodies. Scale bar, 20 μm.

B Quantification of (i) phalloidin and (ii) APPL1 signal intensity in cells serum starved or treated with 10 μM LPA for 5 min. Data are the mean of $n = 3$ independent experiments. Paired t-test $P = 0.0974$ (i) and $P = 0.1481$ (ii). Error bars indicate SEM.

C Quantification of (i) phalloidin and (ii) APPL1 signal intensity in GFP or GFP-LARG GEF transfected cells. Graph depicts mean from $n = 4$ independent experiments. Significance was calculated using a one-sample t-test. *$P < 0.05$, **$P < 0.01$. Error bars indicate SEM.

D Image sequences from spinning disc confocal microscope showing mCherry-APPL1 (red) and BFP-LifeAct (green) in mock (top row) or GFP-LARG GEF (bottom row) transfected HeLa cells. The motility of selected endosomes over time is highlighted by the arrowheads. Scale bar, 10 μm; 0.5 s/frame (see also Movie EV1).

E Confocal microscope images of HeLa cells transfected with GFP-RHOA Q63L (top row), GFP-RHOB Q63L (middle row) and GFP-RHOC Q63L (bottom row). Cells were immunostained with an APPL1 antibody (red) and labelled with phalloidin to visualise actin (green). Scale bar, 20 μm.

© 2018 The Authors

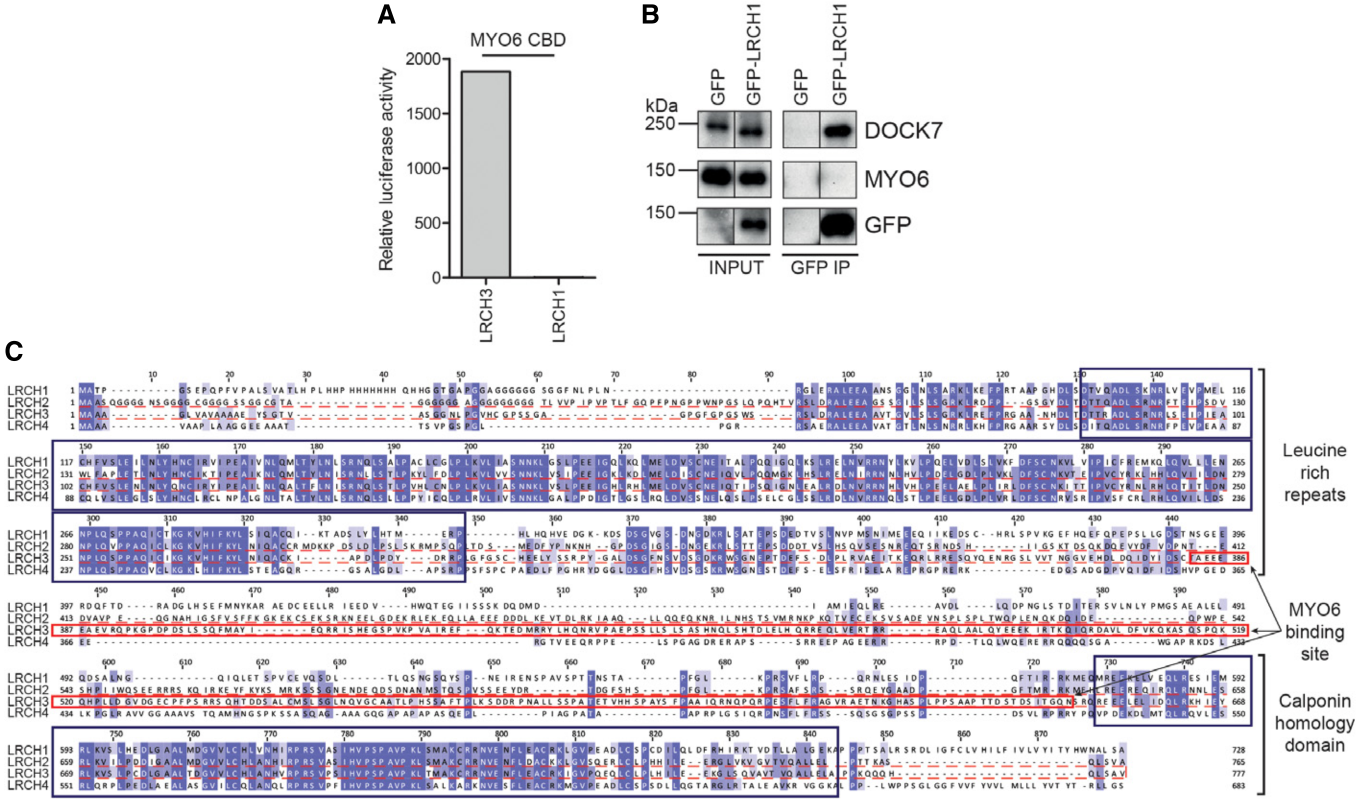

Figure EV4. LRCH1 is not a MYO6 binding partner.

A The mammalian two-hybrid assay was used to test binding of full-length LRCH3 and LRCH1 to wild-type MYO6 tail. Graph shows the mean relative luciferase activity from a single representative experiment.

B GFP nanobody immunoprecipitates from HEK293T cells transfected with GFP and full-length GFP-LRCH1 were analysed by Western blot with the indicated antibodies (GFP control IP same as Fig 5C).

C Sequence alignment of the LRCH proteins. Boxes highlight the highly conserved leucine-rich repeats and calponin homology domains (blue) and the unconserved MYO6 binding site in LRCH3 (red).

 © 2018 The Authors

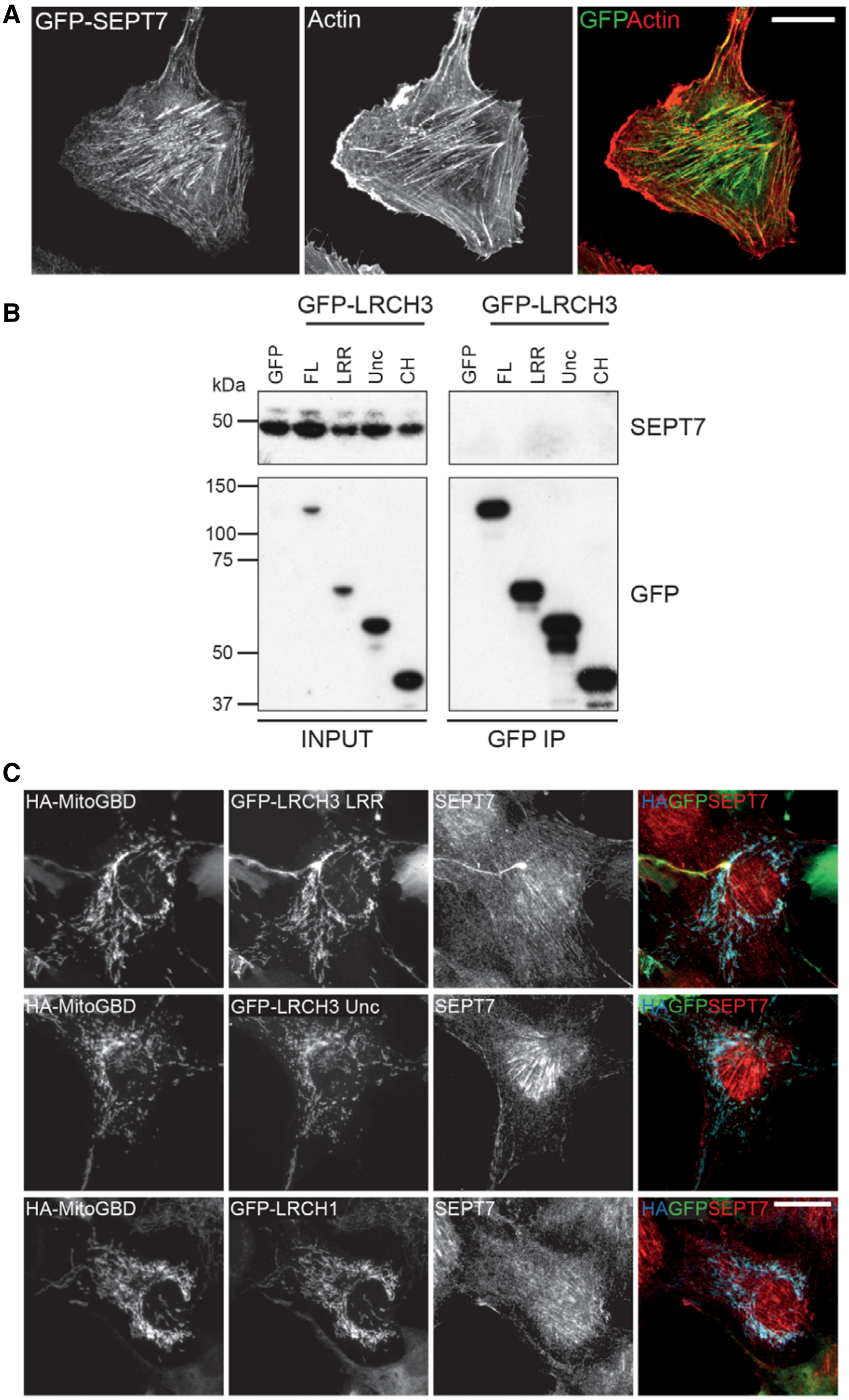

Figure EV5. The DISP complex regulates septin organisation.

A Confocal microscope images of RPE cells stably expressing GFP-SEPT7 and untransfected and immunostained with a GFP (green) antibody and labelled with phalloidin to visualise actin (red). Scale bar, 20 μm.

B GFP nanobody immunoprecipitates from HEK293T cells transfected with GFP, full-length GFP-LRCH3 and GFP-LRCH3 fragments corresponding to amino acids 1–382 (LRR), 383–648 (Unc) or 649–777 (CH). Samples were analysed by Western blot with the indicated antibodies (same IP as Fig 5C).

C Widefield microscope images of RPE cells transfected with HA-MitoGBD and GFP-LRCH3 fragments corresponding to amino acids 1–382 (LRR; top row) and 383–648 (Unc; middle row) or full-length GFP-LRCH1 (bottom row). Cells were immunostained with HA (blue), GFP (green) and SEPT7 (red) antibodies. Scale bar, 20 μm.

© 2018 The Authors
