# Supplementary figures and images for: The MYO6 interactome reveals adaptor complexes coordinating early endosome and cytoskeletal dynamics
Source: EMBO Rep. 2018 Feb 21;19(4):e44884. doi: 10.15252/embr.201744884 (PMC5891429; doi:10.15252/embr.201744884)

...FUJI-HRC-(SAFETY)...

...FUJI-H

TO 18.2.16

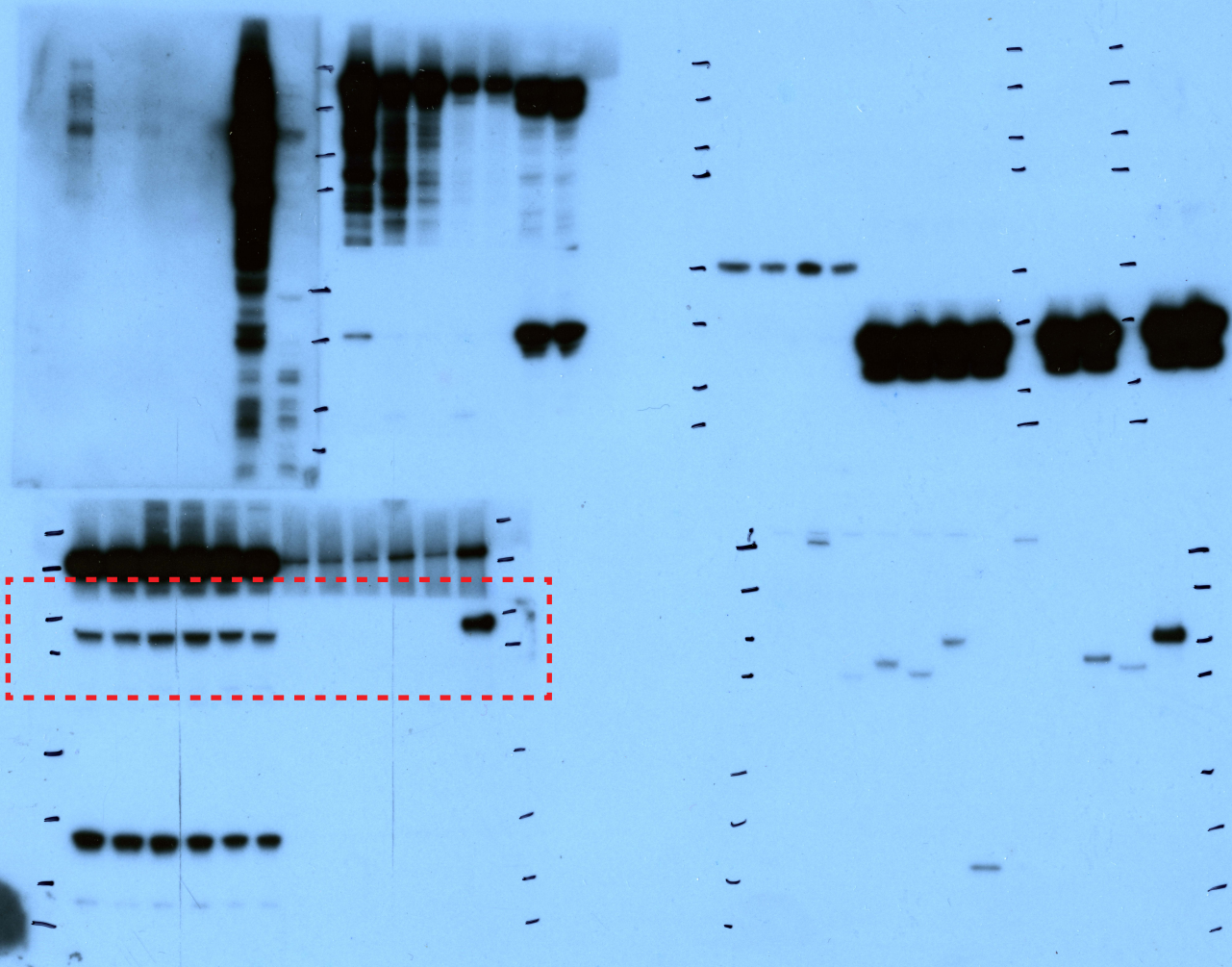

Supplement: Supplementary file 4 — Source Data for Figure 5 [file EMBR-19-e44884-s003.pdf]
