# Supplementary material for: The MYO6 interactome reveals adaptor complexes coordinating early endosome and cytoskeletal dynamics
Source: EMBO Rep. 2018 Feb 21;19(4):e44884. doi: 10.15252/embr.201744884 (PMC5891429; doi:10.15252/embr.201744884)
Supplement: Supplementary file 3 — Review Process File [file EMBR-19-e44884-s004.pdf]

EMBO
reports

The MYO6 interactome reveals adaptor complexes coordinating early endosome and cytoskeletal dynamics

Thomas O'Loughlin, Thomas A Masters, Folma Buss

Review timeline:

Submission date:	24 July 2017
Editorial Decision:	23 August 2017
Revision received:	21 November 2017
Editorial Decision:	14 December 2017
Revision received:	17 January 2018
Accepted:	30 January 2018

Editor: Achim Breiling/ Martina Rembold

Transaction Report:

(Note: With the exception of the correction of typographical or spelling errors that could be a source of ambiguity, letters and reports are not edited. The original formatting of letters and referee reports may not be reflected in this compilation.)

1st Editorial Decision 23 August 2017

Thank you for the submission of your research manuscript to our journal. We have now received the full set of referee reports that is copied below.

As you will see, the referees acknowledge the potential interest of the findings and consider the BioID data for MYO6 largely convincing. However, all referees also suggest several experiments to strengthen the data and conclusions on the further characterization of the novel protein complexes CART and DISP or suggest to tone down the conclusions accordingly. Moreover, all control experiments need to be provided and the experimental details for Fig4 and EV3 added.

Given these constructive comments, we would like to invite you to revise your manuscript with the understanding that the referee concerns (as detailed above and in their reports) must be fully addressed and their suggestions taken on board. Please address all referee concerns in a complete point-by-point response. Acceptance of the manuscript will depend on a positive outcome of a second round of review. It is EMBO reports policy to allow a single round of revision only and acceptance or rejection of the manuscript will therefore depend on the completeness of your responses included in the next, final version of the manuscript.

Revised manuscripts should be submitted within three months of a request for revision; they will otherwise be treated as new submissions. Please contact us if a 3-months time frame is not sufficient for the revisions so that we can discuss the revisions further.

Supplementary/additional data: The Expanded View format, which will be displayed in the main HTML of the paper in a collapsible format, has replaced the Supplementary information. You can submit up to 5 images as Expanded View. Please follow the nomenclature Figure EV1, Figure EV2 etc. The figure legend for these should be included in the main manuscript document file in a section called Expanded View Figure Legends after the main Figure Legends section. Additional Supplementary material should be supplied as a single pdf labeled Appendix. The Appendix

includes a table of content on the first page, all figures and their legends. Please follow the nomenclature Appendix Figure Sx throughout the text and also label the figures according to this nomenclature. For more details please refer to our guide to authors.

Regarding data quantification, please ensure to specify the number "n" for how many independent experiments were performed, the bars and error bars (e.g. SEM, SD) and the test used to calculate p-values in the respective figure legends. Please also include scale bars in all microscopy images.

We now strongly encourage the publication of original source data with the aim of making primary data more accessible and transparent to the reader. The source data will be published in a separate source data file online along with the accepted manuscript and will be linked to the relevant figure. If you would like to use this opportunity, please submit the source data (for example scans of entire gels or blots, data points of graphs in an excel sheet, additional images, etc.) of your key experiments together with the revised manuscript. Please include size markers for scans of entire gels, label the scans with figure and panel number, and send one PDF file per figure or per figure panel.

We would also welcome the submission of cover suggestions, or motifs to be used by our Graphics Illustrator in designing a cover.

As part of the EMBO publication's Transparent Editorial Process, EMBO reports publishes online a Review Process File to accompany accepted manuscripts. This File will be published in conjunction with your paper and will include the referee reports, your point-by-point response and all pertinent correspondence relating to the manuscript.

You are able to opt out of this by letting the editorial office know (emboreports@embo.org). If you do opt out, the Review Process File link will point to the following statement: "No Review Process File is available with this article, as the authors have chosen not to make the review process public in this case."

I look forward to seeing a revised version of your manuscript when it is ready. Please let me know if you have questions or comments regarding the revision.

****
REFEREE REPORTS

Referee #1:

This manuscript describes the use of a proximity-labelling, mass spectrometry approach, BioID, to identify near-neighbours of myosin 6. The authors identify a series of new neighbours/interactors, and perform further BioID with some of these components. In doing so, they identify and partially characterise several new myosin 6- associated complexes. Importantly, two of the complexes contain RHO GEFs that regulate the actin or septin cytoskeleton, meaning that MYO6-associated components can potentially regulate cytoskeletal and endosome dynamics in a co-ordinated fashion. Overall, this is a convincing study that is very well presented and executed and adds considerably to our understanding of MYO6 and its interactors. I have only minor criticisms.

1. A BioID study of dynein has been published (Redwine et al. (2017). The human cytoplasmic dynein interactome reveals novel activators of motility. eLife, 6. http://doi.org/10.7554/eLife.28257), so this is the first BioID investigation of a myosin, not of a motor. The text needs to be changed in several places to take account of that.
2. It would be helpful to provide a brief description of the BioID approach in the introduction or results for readers who don't already know it, and references should be provided.
3. The data presented show that the BirA*-MYO6 localises as expected in the RPE cell line, but it would be useful to know if it is fully functional. Does it rescue a knockdown, for example? Also, what is the relative level of expression of the tagged version in comparison to the endogenous protein?
4. On page 7, the authors state that CADR10 is a high-confidence MYO6 interactor that was present in the GIPC1 data set, however it does not appear in the list in Fig. 3A.
5. The authors use an antibody to SH3BP4 for IF and immunoblotting, but only show a knockdown

control for blotting. Since the IF labelling is fairly non-descript, it would be good to show that knockdown also removes that staining.
6. Do the authors have a positive control for the septin IP data in Fig. EV5A?
7. On p11 the authors state that over-expression of LRCH3 led to the displacement of septins from the actin cytoskeleton, as they clearly take up unusual ring shaped structures. However, actin labelling is not shown, so it is formally possible that actin is rearranged too. It would be useful to show actin labelling in parallel as a control.
8. The enhancement of septin rings when LRCH3 and the DOCK7 DHR2 domain are co-expressed is dramatic. Is this MYO6 dependent?
9. The authors coin a new name for one of the complexes they identify: CART. One concern with this is that it is very similar to CARTS, which is also a trafficking-related name (Wakana et al. (2012). A new class of carriers that transport selective cargo from the trans Golgi network to the cell surface. EMBO Journal, 31, 3976-3990).
10. The methods are clearly defined, except that there is no description of the HA-surface labelling protocol used for the LPA stimulation experiments. This means it is hard to understand exactly what has been done in Fig. 4 and EV3.
11. It is hard to see the difference between the fine and thicker grey lines in Fig. 2. What is a force-directly layout?
12. How many cells were analysed in Fig. 4D?

Referee #2:

This is an ambitious effort using BioID to identify proteins that interact directly or indirectly with myosin-6 in human cells. After a first round of BioID, the authors confirmed the interactions with BioID from the initial partners and IP's to expand the network and verify the partners. The text and figures are clear and easy to follow. This is a valuable addition to the literature, but can be improved by attention to the following points.

Page 3: It would be helpful to spell out or define in a short phrase the meaning of "DAB2, GIPC1, TOM1, LMTK2, OPTN, TAX1BP1 and NDP52". What are the meanings of "RRL and WWY?"

Page 5: Why fuse BirA to only the tail domain of Myo6? Surely the head domain has some influence on the targeting of Myo6 in cells. The text should have brief explanation of how BioID works, including its limitations. I think that the figure for "SILAC-based approach" is Fig. 1D not 1C.

Page 8: Reword "Together these data show".... The micrographs are so small that it is difficult to appreciate that "actin structures" are filopodia at the cell surface (Fig. 3D)." Consider showing higher magnification details. Also the concept of "filopodia protruding from the endosome surface" is difficult to understand. Is filopodium an appropriate word to describe these structures?

Page 9: It is essentially impossible to confirm the "colocalisation of APPL1 with actin upon LPA stimulation (Fig. 4C and Fig EV3B)" because the image is so small (even when enlarged 4 times). The used of dark blue for actin does not help. The text might explain how is Myo6 related to the conclusion "CART complex is an actin regulatory module, which functions downstream of GPCRs such as LPAR1 to drive RHO-mediated actin reorganisation at the early endosome surface, regulating organelle positioning and motility."

Page 12: Overall the data support the authors' conclusions, but they may go a bit too far with "We show that the GEF activity of LARG or LPAR1-LARG-RHO signalling have profound effects on the positioning and motility of MYO6-GIPC1-positive endosomes." True interesting things happen with the reverse mutant myo6+, but that may not be enough to establish "profound effects". "Actin remodeling" as the output of this pathway in Fig. 4A is appropriately vague, since the mechanisms are not established.

Page 13: "These (septin) filaments are intimately linked to the actin cytoskeleton" may also be an overstatement. The experiment showing overexpression of the DISP complex results in septin rings is only indirect (and possibly misleading) evidence for the DISP complex regulating septins under normal conditions, given that "the precise mechanistic details" are missing.

Page 18: You did not fix the cells with paraformaldehyde. It is a solid, which you converted to formaldehyde to fix the cells.

Referee #3:

The authors perform a proteomic study of the unconventional minus-end directed actin motor Myo6. They identify over 50 new potential interactions using the biotin ligase BirA fused to two different Myo6 constructs. They do further pulldowns and proteomic analysis on some of the prominent hits to confirm and identify networks. The proteomic data are nicely presented and appear to be carefully done. New binding partners for Myo6 give insight into the function of this motor and will open up new areas of study. Unfortunately, the specific studies of the two complexes are less convincing and the data surrounding these studies seems to be somewhat over-interpreted. Specific comments follow:

Major Points

What is the significance of the two different Myo6 constructs CBD-LI and CBD NI used for the pulldowns? They were described as interacting with different populations of vesicles but then no data are shown to address whether they have different binding partners or not?

Pulldowns (Fig 3) do not really provide sufficient evidence to claim that a complex of MYO6, GIPC1, LARG and SH3BP4 exists - just that these proteins cross-interact. Can the authors use a method such as blue native PAGE or gel filtration to show that an actual complex exists? If not, then this needs to be toned down as the interactions may be transient and this complex may not actually be present in cells.

Figure 3E- Are these filopodia? Maybe actin clusters is a better term? How was an actin cluster or filopodium defined? This seems unclear and the data presented are not very convincing.

Figure 4 is not convincing and the importance of the colocalization with actin is not clear. When the cells are stimulated by LPA or LARG, endosomes align along actin fibers and are less mobile in the cytoplasm. But is this just because the cytoplasm is more crowded and so the endosomes are trapped in the stress fibers? Are endosomes normally associated with actin stress fibers? What is the function or reason for this? To me it seems like these data are over-interpreted.

It is pretty unclear from Figure 6 how directly Myo6 is involved with Sept7, as these are all pretty indirect experiments showing that disruption of the actin network that normally holds the Sept7 on stress fibers causes them to instead assemble rings. Maybe again the interpretation just needs qualification and toning down a bit.

1st Revision - authors' response 21 November 2017

Point-by-point response to reviewer's queries:

Reviewer #1

1. A BioID study of dynein has been published (Redwine et al. (2017). The human cytoplasmic dynein interactome reveals novel activators of motility. eLife, 6. http://doi.org/10.7554/eLife.28257, so this is the first BioID investigation of a myosin, not of a motor. The text needs to be changed in several places to take account of that.

We have amended the text in the abstract, the end of the introduction and at the beginning of the discussion, which now states correctly that our study provides the first interactome for a myosin motor protein.

2. It would be helpful to provide a brief description of the BioID approach in the introduction or results for readers who don't already know it, and references should be provided.

A short description of BioID is now included on page 4 of the introduction.

3. The data presented show that the BirA-MYO6 localises as expected in the RPE cell line, but it would be useful to know if it is fully functional. Does it rescue a knockdown, for example? Also, what is the relative level of expression of the tagged version in comparison to the endogenous protein?*

In our experiments we only used the C-terminal cargo-binding domain (CBD) of MYO6, which is sufficient for cellular targeting and contains the major protein and lipid binding motifs. As BioID naturally has a limited range of approximately 10 nm (Kim et al., PNAS, 2014), and the full-length MYO6 is between 15-20 nm (Lister et al., EMBO J, 2004), we obtained the highest quality data using only the CBD. We now included a sentence explaining our rationale (the limited reach of BioID) in the results on page 6.

Therefore, beyond targeting and binding to known adaptor proteins, we cannot test whether the CBD is fully functional, as this domain alone will not be able to rescue MYO6-depleted cells. In addition, determining the relative expression of our BirA* construct versus the endogenous is difficult to assess as we don't have antibodies (despite trying to raise one...) which react with both the full-length endogenous protein as well as the CBD used in our study. Furthermore, due to the differences in size or other properties between endogenous MYO6 (~150 kDa) and BirA*-MYO6 CBD (~62 kDa) we would argue any such comparison would be difficult to interpret. For example, differences in membrane transfer due to differing size/charge or possible auto-inhibitory back folding of the endogenous MYO6 altering antigen availability. We are satisfied that the BirA* constructs localised clearly to their relevant compartments which appeared morphologically normal.

4. On page 7, the authors state that CADR10 is a high-confidence MYO6 interactor that was present in the GIPC1 data set, however it does not appear in the list in Fig. 3A.

This oversight has been amended and CARD10 is now present in the list in Fig. 3A.

5. The authors use an antibody to SH3BP4 for IF and immunoblotting, but only show a knockdown control for blotting. Since the IF labelling is fairly non-descript, it would be good to show that knockdown also removes that staining.

A figure showing immunofluorescence staining of mock and SH3BP4 siRNA treated cells with the SH3BP4 antibody is now included in figure 3D, replacing the previous image. We have also included images through the Z-stack to highlight the specificity of the actin labelling.

6. Do the authors have a positive control for the septin IP data in Fig. EV5A?

GFP-LRCH3 and several truncation mutants were immunoprecipitated with GFP-nanobodies and the same eluants were blotted for DOCK7, MYO6 and also SEPT7. Our results show that DOCK7 and MYO6 successfully co-immunoprecipitate with LRCH3 (shown in Fig. 5C), however, we were not able to pull down a complex of GFP-LRCH3 and SEPT7 (shown in Fig. EV5B). The positive control for the septin IP from figure EV5A is therefore shown in figure 5C. We have highlighted the fact that the same IP was blotted for DOCK7, MYO6 as well as SEPT7 in the results on page 12.

7. On p11 the authors state that over-expression of LRCH3 led to the displacement of septins from the actin cytoskeleton, as they clearly take up unusual ring shaped structures. However, actin labelling is not shown, so it is formally possible that actin is rearranged too. It would be useful to show actin labelling in parallel as a control.

Figure 6B has now been modified and structured illumination microscope images are included showing labelling of the actin cytoskeleton. In addition, we have added a sentence to highlight this: "Overexpression of LRCH3 alone does not lead to any obvious changes in actin filament organisation but led to the displacement of septins from actin filaments ….."

8. The enhancement of septin rings when LRCH3 and the DOCK7 DHR2 domain are co-expressed is dramatic. Is this MYO6 dependent?

The reorganisation of the septin cytoskeleton induced by overexpression of LRCH3 and DOCK7 DHR2 does not appear to depend on MYO6 and is unaffected in both MYO6 siRNA KD cells and MYO6 CRISPR KO cells. However, MYO6 may play a role in triggering septin reorganization in a spatial and temporal restricted manner, which is masked by overexpression of LRCH3 and the DHR2 domain of DOCK7.

9. The authors coin a new name for one of the complexes they identify: CART. One concern with this is that it is very similar to CARTS, which is also a trafficking-related name (Wakana et al. (2012). A new class of carriers that transport selective cargo from the trans Golgi network to the cell surface. EMBO Journal, 31, 3976-3990).

Sorry we have missed the overlap with the CARTS and have renamed our complex to LIFT (**L**ARG-**I**nduced **F**-actin for **T**ethering).

10. The methods are clearly defined, except that there is no description of the HA-surface labelling protocol used for the LPA stimulation experiments. This means it is hard to understand exactly what has been done in Fig. 4 and EV3.

The missing method has now been included on page 20 in the Materials and Methods.

11. It is hard to see the difference between the fine and thicker grey lines in Fig. 2. What is a force-directly layout?

The fine lines have now been changed to red.

A force-directed layout is a type of graph drawing algorithm, which assigns forces (which can be attractive or repulsive) to the nodes and edges in the plot and then simulates their movement to create the lowest energy state. This leads to, for example, minimisation of overlapping edges or the clustering of nodes in regions of highly interconnected data. Details of the algorithms can be found within the Cytoscape software manual or, alternatively, an overview of force-directed layout algorithms generally can be viewed here: https://en.wikipedia.org/wiki/Force-directed_graph_drawing.

12. How many cells were analysed in Fig. 4D?

Figure 4D depicts the mean Pearson's correlation coefficient calculated from n=4 independent experiments. In each experiment 1-7 cells per field were quantified from ≥7 randomly selected fields giving a total of 106 mock cells & 137 GFP-GEF expressing cells. We have also added clarifying comments to the legends of all figures containing quantification.

<u>Reviewer #2</u>

Page 3: It would be helpful to spell out or define in a short phrase the meaning of "DAB2, GIPC1, TOM1, LMTK2, OPTN, TAX1BP1 and NDP52". What are the meanings of "RRL and WWY?"

These protein names are now spelled out on page 3 of the introduction. The RRL and WWY motif is a short amino acid sequence, named after their amino acid composition. Again, this has been highlighted in the text on page 3.

2. Page 5: Why fuse BirA to only the tail domain of Myo6? Surely the head domain has some influence on the targeting of Myo6 in cells. The text should have brief explanation of how BioID works, including its limitations. I think that the figure for "SILAC-based approach" is Fig. 1D not 1C.

We now have included a short description of BioID in the introduction on page 4 reading: "This method utilises a promiscuous variant of the *E. Coli* biotin ligase - BirA* - which, rather than modifying a single defined substrate, releases a reactive biotin intermediate (biotinoyl-5'-AMP) into its surroundings [24] . Subsequently, this biotinoyl-5'-AMP intermediate can react with primary

amines in proximal proteins which can then be isolated using the high affinity interaction between the newly generated biotin tag and streptavidin. As the biotin is covalently attached to its target this permits lysis and purification under harsh, denaturing conditions while still preserving weak or transient interactions."

In addition, we have highlighted the limitations in reach of the enzyme and therefore the need to fuse the BirA* enzyme to defined protein domains in our result section on page 6: "As BioID has a limited labelling radius, we used the truncated CBD in our experiments which is sufficient for adaptor and lipid binding and therefore subcellular targeting. Attaching BirA* at the N-terminus of the full-length protein (before the motor domain) largely failed to identify cargo interactions at the C-terminus, presumably due to the limited range of the biotinylation reaction (data not shown)."

The figure reference in the text has now been corrected.

3. Page 8: Reword "Together these data show".... The micrographs are so small that it is difficult to appreciate that "actin structures" are filopodia at the cell surface (Fig. 3D)." Consider showing higher magnification details. Is filopodium an appropriate word to describe these structures?

The text has been corrected.

We appreciate that it is very difficult to determine whether the SH3PB4-positive actin structures are filopodia protruding from the cell surface and have now included images through the Z-stack to clarify the colocalisation in this dimension. In addition, we have included immunofluorescence images from SH3BP4-depleted cells to further highlight the specificity of this staining.

Also the concept of "filopodia protruding from the endosome surface" is difficult to understand.

The term "filopodia protruding from the endosome surface" is not very precise and now has been change to "filopodia protruding from the cell surface above a cortical cluster of endosomes" on page 9 of our results section.

Is filopodium an appropriate word to describe these structures?
We have characterised these spike-like actin protrusions induced by MYO6+ in great detail in our recent PNAS paper (Masters et al., 2017) which is referenced in the manuscript. In that study we show these structures contain both myosin X and fascin, classic marker proteins for filopodia.

4. Page 9: It is essentially impossible to confirm the "colocalisation of APPL1 with actin upon LPA stimulation (Fig. 4C and Fig EV3B)" because the image is so small (even when enlarged 4 times). The used of dark blue for actin does not help.

We hope the submission of high quality eps/tiff files over the low quality jpegs of the initial submission will help to clarify this point. To make the colocalisation of APPL1 and actin more obvious, we have changed the colour of the actin channel to green as suggested and now show several images of enlarged areas. However, these are only select examples of the results quantified in three independent experiments (n=3) on more than 80 different cells.

The text might explain how is Myo6 related to the conclusion "CART complex is an actin regulatory module, which functions downstream of GPCRs such as LPAR1 to drive RHO-mediated actin reorganisation at the early endosome surface, regulating organelle positioning and motility."

We have recently shown (Masters et al. Cell Reports 2017) that MYO6 mediates association of these endosomes with cortical actin filaments. Depletion of MYO6 affects endosome localisation and leads to displacement of these endosomes from the cell cortex into the perinuclear space. Furthermore, we recently demonstrated (Masters et al. PNAS 2017) that expression of the plus end directed MYO6+, thus reversing the direction of MYO6, leads to accumulation and clustering of APPL1 endosomes at the cell at the base of filopodia.

In summary, our previous studies clearly indicate an important role of MYO6 in tethering APPL1 endosomes to cortical actin filaments and thus our results now show that MYO6 might coordinate LARG-induced actin rearrangements in order to regulate endosome position. We have amended the

text in the result section accordingly to emphasise these published findings and to further explain the role of MYO6 in endosome positioning along actin filaments. The new text now reads: "Taken together this data suggests that the LIFT complex is an actin regulatory module, which functions downstream of GPCRs such as LPAR1 to drive RHO-mediated actin reorganisation to regulate endosome positioning and motility. These results support, and may provide the molecular mechanism for, our recent finding that MYO6 mediates association of APPL1 endosomes with cortical actin filaments [33], [34]. Depletion of MYO6 or expression of the reverse MYO6+ affects endosome localisation in the cell cortex. In this way MYO6 could either regulate endosome position directly through organelle tethering to actin filaments or indirectly through reorganization of the actin cytoskeleton involving recruitment of RhoGEFs such as LARG."

5. Page 12: Overall the data support the authors' conclusions, but they may go a bit too far with "We show that the GEF activity of LARG or LPAR1-LARG-RHO signalling have profound effects on the positioning and motility of MYO6-GIPC1-positive endosomes." True interesting things happen with the reverse mutant myo6+, but that may not be enough to establish "profound effects". "Actin remodeling" as the output of this pathway in Fig. 4A is appropriately vague, since the mechanisms are not established.

Our conclusions have been toned down and the text now reads: "We show that the GEF activity of LARG or LPAR1-LARG-RHO signalling can affect the positioning and motility of MYO6-GIPC1-positive endosomes."

6. Page 13: "These (septin) filaments are intimately linked to the actin cytoskeleton" may also be an overstatement…

This statement is based on published observation and our immunofluorescence images shown in figure EV5, which clearly shows alignment of SEPT7 along actin filaments. However, we have changed the wording and the new text now reads: "These filaments have been linked to the actin cytoskeleton by colocalisation and observations that perturbation of the actin cytoskeleton causes the formation of septin rings or, conversely, septin depletion triggers the loss of actin [33], [38], [39]."

… The experiment showing overexpression of the DISP complex results in septin rings is only indirect (and possibly misleading) evidence for the DISP complex regulating septins under normal conditions, given that "the precise mechanistic details" are missing.

Our results are based on overexpression of the DOCK7 GEF, which in the presence of LRCH3 induces a dramatic reorganisation of the septin cytoskeleton without inducing a visible change in actin organisation. So far very few proteins have been identified that induce such an obvious change in septin localisation and which can be assessed and quantified. We agree, at present we cannot present any insight into "precise mechanistic details", however, the regulation of septin function is likely to involve a number of different mechanisms and regulators dependent of the different cellular processes that require septin activity. We therefore feel that it is beyond the scope of this project to determine the details of septin cytoskeleton regulation, however, our discovery of a RhoGEF involved in this process is important.

7. Page 18: You did not fix the cells with paraformaldehyde. It is a solid, which you converted to formaldehyde to fix the cells.

Sorry, this has been changed now.

Reviewer #3

What is the significance of the two different Myo6 constructs CBD-LI and CBD NI used for the pulldowns? They were described as interacting with different populations of vesicles but then no data are shown to address whether they have different binding partners or not?

A detailed comparative analysis is now included in figure 1 and we have amended the manuscript to discuss these results as follows: "Comparison of the NI and LI showed 39 shared interactions and 16 or 47 specific interactions for the LI and NI isoforms respectively. Many of the known direct

binding partners of MYO6 appear in the shared pool of interactions for the two isoforms (Fig. 1E). This confirms our previous observations that binding of DAB2 and other adaptors is not isoform specific [8], [10], [14], but targeting of the LI isoform to clathrin-coated structures is directed by the large insert [27]. As a result, the LI still appears to show enrichment for CCS proteins such as AP2 subunits, SYNJ1 and PICALM, whereas the NI specific interactions are less well annotated but are likely to link it to diverse cellular localisations and functions".

2. Pulldowns (Fig 3) do not really provide sufficient evidence to claim that a complex of MYO6, GIPC1, LARG and SH3BP4 exists - just that these proteins cross-interact. Can the authors use a method such as blue native PAGE or gel filtration to show that an actual complex exists? If not, then this needs to be toned down as the interactions may be transient and this complex may not actually be present in cells.

We have shown the reciprocity of the interactions between MYO6, GIPC1, LARG and SH3BP4 by multiple methods including BioID (with MYO6, GIPC1 and LARG), immunoprecipitations and mammalian two-hybrid assays. Together, we feel our data provides strong evidence for the presence of this complex. Of course, the referee is correct to point out that the components may not form a stable complex and we agree with the possibility that the complex only forms transiently in living cells. We do not feel we have overstated the stability/transience of this complex in the manuscript but we are happy to change the text and emphasise this point if required.

3. Figure 3E- Are these filopodia? Maybe actin clusters is a better term? How was an actin cluster or filopodium defined? This seems unclear and the data presented are not very convincing.

We have described these actin structures, which are induced by the plus-end directed MYO6 protruding from the cell surface, in great detail in our recent PNAS paper (Masters et al., 2017). We used a range of different markers and show that they are indeed filopodia-like actin protrusions, which contain myosin X and also fascin, proteins associated with filopodia. We inserted the reference (Masters et al. PNAS 2017) in the result section on page 9 to refer to our published characterisation of these MYO6+-induced filopodia.

4. Figure 4 is not convincing and the importance of the colocalization with actin is not clear. When the cells are stimulated by LPA or LARG, endosomes align along actin fibers and are less mobile in the cytoplasm. But is this just because the cytoplasm is more crowded and so the endosomes are trapped in the stress fibers? Are endosomes normally associated with actin stress fibers? What is the function or reason for this? To me it seems like these data are over-interpreted.

We have previously performed a very careful characterisation of the association of different types of endosomes with actin filaments using high-resolution structured illumination microscopy. Our results demonstrate that the APPL1 endosomes that are positive for MYO6 align along actin filament bundles whereas EEA1-positive endosomes, which do not colocalize with MYO6, are surrounded by actin filament patches (Masters et al., Cell Reports 2017). These results indicate that while both APPL1- and EEA1-positive endosomes are associated with actin, the architecture and geometry of these interactions are highly divergent and therefore we feel that it is highly unlikely that "the endosomes are trapped in the stress fibers". We observe the APPL1 endosomes associate with actin filament bundles of different sizes concentrating in the cortical actin network, however, sometimes these endosomes also align along larger actin bundles, which could be termed stress fibers.

In light of our previous published findings that MYO6 has an important role in tethering APPL1 endosomes to cortical actin filaments, we feel that our data is not over-interpreted. Depletion of MYO6 affects endosome localisation and leads to displacement of these endosomes from the cell cortex into the perinuclear space. Furthermore, we recently demonstrated (Masters et al. PNAS 2017) that expression of the plus end directed MYO6+, thus reversing the direction of MYO6, leads to accumulation and clustering of APPL1 endosomes at the base of filopodia.

5. It is pretty unclear from Figure 6 how directly Myo6 is involved with Sept7, as these are all pretty indirect experiments showing that disruption of the actin network that normally holds the Sept7 on stress fibers causes them to instead assemble rings. Maybe again the interpretation just needs qualification and toning down a bit.

We now have amended figure 6 and have included actin staining, which clearly shows that the overexpression of the DOCK7 GEF, in the presence of LRCH3, induces a dramatic reorganisation of the septin cytoskeleton, without inducing a visible change in actin organisation. So DOCK7 and LRCH3 cause a highly specific reorganisation of the septin cytoskeleton, which is not caused by simply depolymerisation of actin filaments.

Unfortunately, so far we are not able to show a direct requirement for MYO6 in septin cytoskeleton regulation, however, as shown in figure 6C, MYO6 is recruited to septin ring structures in the cytoplasm along with LRCH3 and DOCK7.

2nd Editorial Decision 14 December 2017

Thank you for the submission of your revised manuscript to our editorial offices. We have now received the reports from the referees that were asked to re-evaluate your study (you will find enclosed below). As you will see, all three referees now support the publication of your manuscript in EMBO reports.

Before we can proceed with formal acceptance, I have the following final editorial requests:

The manuscript is currently rather long (more than 75000 characters including spaces), even for an article. Thus, I would ask you to shorten the manuscript to around 65000. Especially the figure legends are rather wordy and contain methods information and detailed descriptions of the results, which could be removed if redundant.

For the references, please use 'et al' for those references with ten or more authors. Please also list the references with simple numbering (without the square brackets) in the reference section. Square brackets should only be used for the call outs. See also:
http://embor.embopress.org/authorguide#referencesformat

Please add a reference or an accession number for the data deposited at the PRIDE proteomics data repository.

Please add the title "Conflict of interest statement" above the conflict of interest statement.

Please rename the movie file as "Movie EV1" and then combine the movie file with a simple text file of the legend in a ZIP archive file, and upload the ZIPped file. Then please update the callout for the movie in the text and remove the legend from the main manuscript text.

Finally, could statistics be provided for the bar diagrams in Figs. 4C and EV3B?

We now strongly encourage the publication of original source data, in particular of Western blots, with the aim of making primary data more accessible and transparent to the reader. The source data will be published in a separate source data file online along with the accepted manuscript and will be linked to the relevant figure. If you would like to use this opportunity, please submit the source data (for example scans of entire gels or blots, data points of graphs in an excel sheet, additional images, etc.) of your key experiments together with the revised manuscript. Please include size markers for scans of entire gels, label the scans with figure and panel number, and send one PDF file per figure.

In addition I would need from you:
- a short, two-sentence summary of the manuscript
- two to three bullet points highlighting the key findings of your study
- a schematic summary figure (in jpeg or tiff format with the exact width of 550 pixels and a height of about 400 pixels) that can be used as visual synopsis on our website.

I look forward to seeing the final revised version of your manuscript when it is ready. Please let me know if you have questions or comments regarding the revision.

REFEREE REPORTS

Referee #1:

The authors have dealt successfully with all my comments and I think the manuscript has been improved and should be published in EMBO Reports.

Referee #2:

The authors made a serious effort to respond to all of my concerns raised in the first round of review. I am satisfied with their responses and the revised paper.

Referee #3:

The authors have provided additional clarifications and data where requested. All of my major points have been addressed and I think that this study will make an interesting addition to our understanding of how MYO6 interacts with multiple proteins to organise the cytoskeleton and coordinate trafficking.

2nd Revision - authors' response 17 January 2018

As suggested in your email from the 14th December 2017, we have amended our manuscript as follows:

1. We have reduced our total character count below 70,000. As discussed by email earlier this week we are happy to reduce the character count further by moving more detailed methods to supplementary information if required.

2. The references have been amended and we have used 'et al' for those references with ten or more authors.

3. The accession number for the PRIDE proteomics data repository has now been included.

4. We now have added the title "Conflict of interest statement" above the conflict of interest statement.

5. The ZIP file with the movie is now uploaded separately.

6. Finally, the text file of the manuscript, TIFs of the figures, a short, two-sentence summary of the manuscript, two to three bullet points highlighting the key findings of our study and a schematic summary figure are now included.

© European Molecular Biology Organization

EMBO PRESS

YOU MUST COMPLETE ALL CELLS WITH A PINK BACKGROUND ➔

PLEASE NOTE THAT THIS CHECKLIST WILL BE PUBLISHED ALONGSIDE YOUR PAPER

Corresponding Author Name: Dr. Folma Buss

Journal Submitted to: EMBO Reports

Manuscript Number: EMBOR-2017-44884-T

Reporting Checklist For Life Sciences Articles (Rev. June 2017)

This checklist is used to ensure good reporting standards and to improve the reproducibility of published results. These guidelines are consistent with the Principles and Guidelines for Reporting Preclinical Research issued by the NIH in 2014. Please follow the journal's authorship guidelines in preparing your manuscript.

A- Figures
1. Data
The data shown in figures should satisfy the following conditions:

- ➔ the data were obtained and processed according to the field's best practice and are presented to reflect the results of the experiments in an accurate and unbiased manner.
- ➔ figure panels include only data points, measurements or observations that can be compared to each other in a scientifically meaningful way.
- ➔ graphs include clearly labeled error bars for independent experiments and sample sizes. Unless justified, error bars should not be shown for technical replicates.
- ➔ if n< 5, the individual data points from each experiment should be plotted and any statistical test employed should be justified
- ➔ Source Data should be included to report the data underlying graphs. Please follow the guidelines set out in the author ship guidelines on Data Presentation.

2. Captions

Each figure caption should contain the following information, for each panel where they are relevant:

- ➔ a specification of the experimental system investigated (eg cell line, species name).
- ➔ the assay(s) and method(s) used to carry out the reported observations and measurements
- ➔ an explicit mention of the biological and chemical entity(ies) that are being measured.
- ➔ an explicit mention of the biological and chemical entity(ies) that are altered/varied/perturbed in a controlled manner.

- ➔ the exact sample size (n) for each experimental group/condition, given as a number, not a range;
- ➔ a description of the sample collection allowing the reader to understand whether the samples represent technical or biological replicates (including how many animals, litters, cultures, etc.).
- ➔ a statement of how many times the experiment shown was independently replicated in the laboratory.
- ➔ definitions of statistical methods and measures:
  - common tests, such as t-test (please specify whether paired vs. unpaired), simple χ2 tests, Wilcoxon and Mann-Whitney tests, can be unambiguously identified by name only, but more complex techniques should be described in the methods section;
  - are tests one-sided or two-sided?
  - are there adjustments for multiple comparisons?
  - exact statistical test results, e.g., P values = x but not P values < x;
  - definition of 'center values' as median or average;
  - definition of error bars as s.d. or s.e.m.

Any descriptions too long for the figure legend should be included in the methods section and/or with the source data.

In the pink boxes below, please ensure that the answers to the following questions are reported in the manuscript itself. Every question should be answered. If the question is not relevant to your research, please write NA (non applicable). We encourage you to include a specific subsection in the methods section for statistics, reagents, animal models and human subjects.

USEFUL LINKS FOR COMPLETING THIS FORM

http://www.antibodypedia.com
http://1degreebio.org
http://www.equator-network.org/reporting-guidelines/improving-bioscience-research-repo
http://grants.nih.gov/grants/olaw/olaw.htm
http://www.mrc.ac.uk/Ourresearch/Ethicsresearchguidance/Useofanimals/index.htm
http://ClinicalTrials.gov
http://www.consort-statement.org
http://www.consort-statement.org/checklists/view/32-consort/66-title
http://www.equator-network.org/reporting-guidelines/reporting-recommendations-for-tun
http://datadryad.org
http://figshare.com
http://www.ncbi.nlm.nih.gov/gap
http://www.ebi.ac.uk/ega
http://biomodels.net/
http://biomodels.net/miriam/
http://jjj.biochem.sun.ac.za
http://oba.od.nih.gov/biosecurity/biosecurity_documents.html
http://www.selectagents.gov/

B- Statistics and general methods

	Please fill out these boxes ➔ (Do not worry if you cannot see all your text once you press return)
1.a. How was the sample size chosen to ensure adequate power to detect a pre-specified effect size?	≥n=3 independent replicates were performed for each experiment
1.b. For animal studies, include a statement about sample size estimate even if no statistical methods were used.	n/a
2. Describe inclusion/exclusion criteria if samples or animals were excluded from the analysis. Were the criteria pre-established?	n/a
3. Were any steps taken to minimize the effects of subjective bias when allocating animals/samples to treatment (e.g. randomization procedure)? If yes, please describe.	n/a
For animal studies, include a statement about randomization even if no randomization was used.	n/a
4.a. Were any steps taken to minimize the effects of subjective bias during group allocation or/and when assessing results (e.g. blinding of the investigator)? If yes please describe.	n/a
4.b. For animal studies, include a statement about blinding even if no blinding was done	n/a
5. For every figure, are statistical tests justified as appropriate?	Yes, tests are outlined in figure legends
Do the data meet the assumptions of the tests (e.g., normal distribution)? Describe any methods used to assess it.	Results of tests are described in the manuscript
Is there an estimate of variation within each group of data?	Yes, SEM values are provided
Is the variance similar between the groups that are being statistically compared?	Yes

C- Reagents

6. To show that antibodies were profiled for use in the system under study (assay and species), provide a citation, catalog number and/or clone number, supplementary information or reference to an antibody validation profile. e.g., Antibodypedia (see link list at top right), 1DegreeBio (see link list at top right).	Antibody catalogue numbers are provided in the materials and methods under the subheading "antibodies and reagents". In-house validation of the antibodies is provided throughout the manuscript in main and expanded view figures
7. Identify the source of cell lines and report if they were recently authenticated (e.g., by STR profiling) and tested for mycoplasma contamination.	HEK293T & RPE-1 cells were originally sourced from the ATCC (CRL-3216 & hTERT-RPE-1 CRL-4000). HeLa M cells were described in Tiwari et al., EMBO J. 1987. Cells were regularly assessed for morphology and mycoplasma by microscopy.

* for all hyperlinks, please see the table at the top right of the document

D- Animal Models

8. Report species, strain, gender, age of animals and genetic modification status where applicable. Please detail housing and husbandry conditions and the source of animals.	n/a
9. For experiments involving live vertebrates, include a statement of compliance with ethical regulations and identify the committee(s) approving the experiments.	n/a
10. We recommend consulting the ARRIVE guidelines (see link list at top right) (PLoS Biol. 8(6), e1000412, 2010) to ensure that other relevant aspects of animal studies are adequately reported. See author guidelines, under 'Reporting Guidelines'. See also: NIH (see link list at top right) and MRC (see link list at top right) recommendations. Please confirm compliance.	n/a

E- Human Subjects

11. Identify the committee(s) approving the study protocol.	n/a
12. Include a statement confirming that informed consent was obtained from all subjects and that the experiments conformed to the principles set out in the WMA Declaration of Helsinki and the Department of Health and Human Services Belmont Report.	n/a
13. For publication of patient photos, include a statement confirming that consent to publish was obtained.	n/a
14. Report any restrictions on the availability (and/or on the use) of human data or samples.	n/a
15. Report the clinical trial registration number (at ClinicalTrials.gov or equivalent), where applicable.	n/a
16. For phase II and III randomized controlled trials, please refer to the CONSORT flow diagram (see link list at top right) and submit the CONSORT checklist (see link list at top right) with your submission. See author guidelines, under 'Reporting Guidelines'. Please confirm you have submitted this list.	n/a
17. For tumor marker prognostic studies, we recommend that you follow the REMARK reporting guidelines (see link list at top right). See author guidelines, under 'Reporting Guidelines'. Please confirm you have followed these guidelines.	n/a

F- Data Accessibility

18: Provide a "Data Availability" section at the end of the Materials & Methods, listing the accession codes for data generated in this study and deposited in a public database (e.g. RNA-Seq data: Gene Expression Omnibus GSE39462, Proteomics data: PRIDE PXD000208 etc.) Please refer to our author guidelines for 'Data Deposition'.<br><br>Data deposition in a public repository is mandatory for:<br>a. Protein, DNA and RNA sequences<br>b. Macromolecular structures<br>c. Crystallographic data for small molecules<br>d. Functional genomics data<br>e. Proteomics and molecular interactions	Proteomics data is deposited with the PRIDE repository and the accession code provided in the materials and methods
19. Deposition is strongly recommended for any datasets that are central and integral to the study; please consider the journal's data policy. If no structured public repository exists for a given data type, we encourage the provision of datasets in the manuscript as a Supplementary Document (see author guidelines under 'Expanded View' or in unstructured repositories such as Dryad (see link list at top right) or Figshare (see link list at top right).	n/a
20. Access to human clinical and genomic datasets should be provided with as few restrictions as possible while respecting ethical obligations to the patients and relevant medical and legal issues. If practically possible and compatible with the individual consent agreement used in the study, such data should be deposited in one of the major public access-controlled repositories such as dbGAP (see link list at top right) or EGA (see link list at top right).	n/a
21. Computational models that are central and integral to a study should be shared without restrictions and provided in a machine-readable form. The relevant accession numbers or links should be provided. When possible, standardized format (SBML, CellML) should be used instead of scripts (e.g. MATLAB). Authors are strongly encouraged to follow the MIRIAM guidelines (see link list at top right) and deposit their model in a public database such as Biomodels (see link list at top right) or JWS Online (see link list at top right). If computer source code is provided with the paper, it should be deposited in a public repository or included in supplementary information.	n/a

G- Dual use research of concern

22. Could your study fall under dual use research restrictions? Please check biosecurity documents (see link list at top right) and list of select agents and toxins (APHIS/CDC) (see link list at top right). According to our biosecurity guidelines, provide a statement only if it could.	n/a
